# Electroacupuncture ameliorates glycolipid metabolism disorder in skeletal muscle of type 2 diabetic rats via modulation of the AMPK/PGC-1α/TFAM signaling pathway

**DOI:** 10.1186/s13098-025-01960-w

**Published:** 2025-12-30

**Authors:** Fang Luo, Junjie Feng, Jumahan Nverjiang, Jiangnan Ye, Chang Liu, Hanhan Chen, Zhuoxuan Li, Qunwen Lu, Wei Zhang, Furong Zhang, Jun Zhu, Chengguo Su

**Affiliations:** 1 Department of Acupuncture-Moxibustion and Tuina, Chengdu University of Traditional Chinese Medicine, Chengdu, 611100 China; 2 College of pharmacy, Chengdu University of Traditional Chinese Medicine, Chengdu, 611100 China; 3 School of Health Preservation and Rehabilitation, Chengdu University of Traditional Chinese Medicine, Chengdu, 611100 China; 4 School of Clinical Medicine, Chengdu University of Traditional Chinese Medicine, Chengdu, 611100 China; 5 School of Health and Rehabilitation, Chengdu University of Traditional Chinese Medicine, Chengdu, 611100 China

**Keywords:** Type 2 diabetes, Electroacupuncture, AMPK/PGC-1α/TFAM pathway, Skeletal muscle, Glucolipid metabolism

## Abstract

**Objective:**

This study aimed to investigate whether electroacupuncture (EA) can regulate skeletal muscle glucose metabolism through the AMPK/PGC-1α/TFAM signaling pathway in a rat model of type 2 diabetes mellitus (T2DM).

**Methods:**

T2DM was induced by feeding rats a high-fat, high-sugar diet followed by intraperitoneal streptozotocin (35 mg/kg). Rats were randomly assigned to five groups: model, EA, EA plus AMPK inhibitor (Compound C, 20 mg/kg, three times weekly), sham acupuncture (tail non-acupoint stimulation), and control. EA was applied at Zusanli, Sanyinjiao, and Weiwanxiashu for 20 min daily, six days per week, for four weeks. Random blood glucose (RBG) and body weight were monitored weekly. After intervention, fasting blood glucose (FBG), triglycerides (TG), low-density lipoprotein cholesterol (LDL-C), fasting insulin (FINS), and C-peptide (C-P) were measured, and HOMA-IR and ISI were calculated based on FBG and FINS. Skeletal muscle morphology was assessed by H&E staining; ATP levels were measured; and AMPK/PGC-1α/TFAM pathway related protein and gene expression were analyzed by Western blotting and RT-PCR.

**Results:**

EA reduced RBG, body weight, FBG, TG, LDL-C, FINS, C-P levels, and HOMA-IR, while improving ISI. Moreover, EA enhances the expression of AMPK, PGC-1α, TFAM, and GLUT 4 at both the protein and mRNA levels, alleviates skeletal muscle cell injury, and increases ATP content in skeletal muscle. These beneficial effects are abolished by co-administration of an AMPK inhibitor.

**Conclusion:**

EA improves glycolipid metabolism and alleviates insulin resistance in T2DM rats, potentially via activation of the AMPK/PGC-1α/TFAM pathway. These effects may be linked to improved skeletal muscle function and glucose utilization. EA shows promise as a therapeutic strategy for T2DM, warranting further investigation into its mechanisms and clinical relevance.

**Supplementary Information:**

The online version contains supplementary material available at 10.1186/s13098-025-01960-w.

## Introduction

Type 2 diabetes mellitus (T2DM) is a metabolic disorder characterized by chronic hyperglycemia and has become one of the most serious diseases in the 21 st century. Persistent hyperglycemia and its associated complications severely compromise patients’ quality of life [31]. China has the largest diabetic population worldwide, and the rapid rise in T2DM cases imposes a substantial economic burden [4]. Currently, there is no definitive cure for T2DM; clinical management primarily relies on pharmacological therapy, dietary regulation, and physical activity to maintain glycemic control and delay complications [39]. However, hypoglycemic agents often cause adverse effects, while poor adherence undermines the effectiveness of lifestyle interventions [72]. Therefore, identifying safe and effective glycemic control strategies has become a key clinical objective.

Acupuncture, a traditional Chinese medical therapy, has emerged as a potential adjunctive approach for regulating glucose metabolism and managing T2DM [18]. The World Health Organization recognizes acupuncture as an important complementary therapy for diabetes [40]. Electroacupuncture (EA) may facilitate glucose uptake, absorption, and transport in skeletal muscle cells, thereby contributing to glycemic regulation in T2DM [61]. Clinical studies have shown [7, 69], Feng et al., [14]) that acupuncture reduces insulin resistance (IR), enhances insulin sensitivity, rapidly lowers blood glucose, and alleviates typical diabetic symptoms. In addition, it may improve hemodynamics [50], repair damaged neural tissue [20], and modulate inflammatory responses [16], contributing to symptom relief and the prevention of complications. However, the mechanisms by which acupuncture improves glucose metabolism in T2DM remain unclear.

IR represents a central mechanism underlying the pathogenesis of T2DM, which leads to abnormal glucose metabolism [63]. IR is characterized by diminished sensitivity and responsiveness of insulin target tissues, resulting in reduced glucose uptake and utilization, which triggers compensatory hyperinsulinemia and glucose metabolism disorders [70]. As an early pathological feature of T2DM, IR is responsible for approximately 24.4% of diabetes cases [54]. Moreover, disturbances in glucose metabolism are frequently associated with dyslipidemia, which inhibits hepatic and muscular glycogen synthesis, promotes gluconeogenesis, elevates circulating free fatty acids (FFAs), and induces pancreatic β-cell apoptosis, thereby suppressing insulin secretion and exacerbating glucose metabolic dysfunction [62]. In Western populations, obese individuals have a 7–12-fold increased risk of developing T2DM compared to those with normal weight, with more than 50% of patients exhibiting overweight or obesity [9, 26]. Therefore, improving IR and correcting lipid abnormalities are critical to the prevention and treatment of glucose metabolic disorders in T2DM.

Skeletal muscle serves as the major energy-metabolizing tissue involved in maintaining glucose homeostasis and is the principal site of IR (Meng et al., [35]). Under normal physiological conditions, it accounts for approximately 85% of whole-body glucose metabolism and 80% of glycogen storage [5]. Studies have shown that nearly 99.8% of diabetic patients exhibit skeletal muscle dysfunction or atrophy, thereby further exacerbating glucose metabolic abnormalities [32]. The function and morphology of skeletal muscle are dependent on ATP production via mitochondrial oxidative phosphorylation (OXPHOS)(Van Huynh et al., [48]. When mitochondrial ATP generation is impaired, the energy demands of the body cannot be met, ultimately resulting in disturbances in glucose metabolism [73]. Therefore, enhancing ATP production and improving skeletal muscle structure and function are essential for mitigating IR and improving glucose metabolic homeostasis.

The AMPK/PGC-1α/TFAM signaling pathway plays a critical role in regulating glucose metabolism in skeletal muscle [71]. AMP-activated protein kinase (AMPK) is a central regulator of cellular homeostasis and is highly sensitive to energy fluctuations in the liver and skeletal muscle [57]. Under hyperglycemic conditions, increased intracellular reactive oxygen species (ROS) and altered AMP/ATP ratios result in decreased AMPK activity, leading to energy imbalance in target tissues and exacerbation of T2DM [53]. Peroxisome proliferator-activated receptor-γ coactivator 1α (PGC-1α), a downstream effector of AMPK, is a key regulator of mitochondrial biogenesis [49]. AMPK activation stimulates PGC-1α, which subsequently upregulates mitochondrial transcription factor A (TFAM), enhancing ATP production and promoting glucose metabolism in skeletal muscle [64]. Additionally, this pathway regulates glucose uptake through modulation of glucose transporter type 4 (GLUT4)(Song [45]. GLUT4, the principal glucose transporter in skeletal muscle, facilitates glucose utilization by translocating from the cytoplasm to the plasma membrane, thereby promoting glycogen synthesis and reducing blood glucose levels [43]. Studies have shown that activation of AMPK or PGC-1α can increase skeletal muscle glucose uptake by more than threefold [41].

Previous studies have demonstrated that acupuncture at the Zusanli acupoint alleviates skeletal muscle atrophy, improves mitochondrial structure and function, and activates AMPK, thereby enhancing OXPHOS [66, 67]. Animal experiments have confirmed that EA activates AMPK and enhances OXPHOS activity, thereby ameliorating metabolic dysfunction in experimental T2DM rats [12]. Moreover, EA increases the expression of PGC-1α and TFAM in the sciatic nerve of T2DM rats, contributing to the protection of damaged neurons [68].

This study focused on the AMPK/PGC-1α/TFAM signaling pathway to investigate the effects of EA on glucose and lipid metabolism as well as IR in a T2DM rat model. The findings elucidate the potential mechanisms by which EA alleviates skeletal muscle glucose metabolic disorders in T2DM. These results provide new insights into the mechanistic basis of EA in the treatment of T2DM and further support its potential as a viable therapeutic option in clinical practice.

## Materials and methods

### Experimental animals

Forty 8-week-old male Sprague–Dawley (SD) rats weighing 200 ± 20 g were purchased from Chengdu Dashuo Laboratory Animal Co., Ltd. The rats underwent a 7-day acclimatization period under controlled conditions (temperature 20–25 °C, relative humidity 55 ± 10%, and a 12-hour light/dark cycle) with ad libitum access to food and water. Bedding, feed, and water were changed daily. All animals were housed in a specific pathogen-free (SPF) facility at the Experimental Center of Chengdu University of Traditional Chinese Medicine and were randomly numbered. All procedures were conducted in accordance with the ethical guidelines for animal experiments approved by the Laboratory Animal Welfare and Ethics Committee of Chengdu University of Traditional Chinese Medicine (Approval No. 2024015).

### T2DM animal model establishment and grouping

After a 1-week acclimatization period, 40 rats were randomly divided into two groups: a control group (*n* = 8) and a modeling group (*n* = 32). The control group was fed a standard diet (18.5% protein, 5.2% fat, 9.4% moisture, 3.5% crude fiber, 6.6% ash, 1.13% calcium, 0.86% phosphorus), whereas the modeling group received a high-fat, high-sugar diet (13.4% protein, 14.3% fat, 23.5% sugar, 9.3% moisture, 2.7% crude fiber, 4.4% ash, 0.83% calcium, 0.71% phosphorus), with free access to water. After 8 weeks of feeding, all rats fasted for 12 h, followed by intraperitoneal injection of streptozotocin (STZ) buffer solution (STZ powder from Sigma, USA) at a dose of 35 mg/kg in the modeling group [65]. Random blood glucose (RBG) levels were measured 24 h post-injection. T2DM modeling was considered successful when RBG exceeded 16.7 mmol/L for seven consecutive days. The control group received an intraperitoneal injection of an equal volume of citrate-sodium citrate buffer (Sigma, USA) at the same concentration, with all other procedures identical to the modeling group. The T2DM model rats were divided into the model, EA, sham EA, and EA + Compound C groups. Throughout the experiment, T2DM model rats were continuously fed the high-fat, high-sugar diet, whereas the control group remained on the standard diet.

### Electroacupuncture intervention

**Fig. 1:**
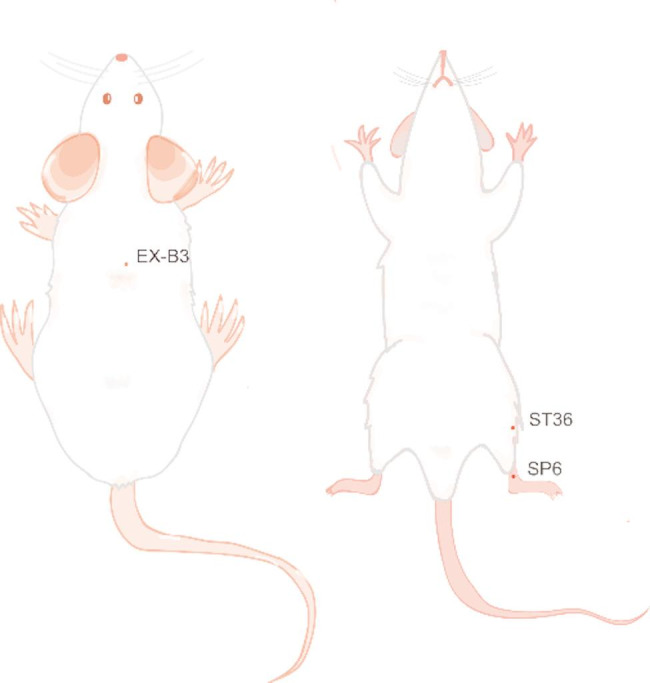
Localization of the acupoints ST36 (Zusanli), SP6 (Sanyinjiao), and EX-B3 (Weiwanxiashu) in rats. ST36, SP6, and EX-B3 represent the international codes for Zusanli, Sanyinjiao, and Weiwanxiashu, respectively

EA was administered to rats at acupoints Zusanli (ST36), Sanyinjiao (SP6), and Weiwanxiashu (EX-B3) (Fig. 1), located according to the “Common Acupoint Names and Locations in Laboratory Animals“ [37]. Sterile Huatuo acupuncture needles (0.25 × 13 mm; Suzhou Medical Supplies Co., Ltd.) were inserted 3–5 mm at each point, connected to an SDZ-II electroacupuncture stimulator (Suzhou Medical Supplies Co., Ltd.). Parameters were set to continuous wave at 15 Hz, with intensity adjusted to induce slight toe twitching. Treatments were applied once daily for 20 min, six consecutive days followed by one rest day, over four weeks.

Sham acupuncture (SA) was performed as described [21], with needles inserted approximately 4 mm deep at three bilateral non-acupoints located ~ 8 mm dorsally from the tail base near the vertebral spinous process, connected to the stimulator using the same parameters and schedule as the EA group.

The EA + Compound C (EA + CC) group received the same EA treatment as the EA group, combined with intraperitoneal injections of Compound C (AMPK inhibitor; Selleckchem, Houston, TX, USA) at 20 mg/kg, administered thrice weekly for 12 doses over four weeks. Compound C inhibits the AMPK factor.

Control and model groups were subjected to the same restraint procedures as the EA group to ensure consistency. All efforts were made to minimize animal pain and discomfort throughout the experiment.

### Collection of materials

After 12 h of fasting at the end of week 4, fasting blood glucose (FBG) was measured. Rats were then anesthetized via intraperitoneal injection of 1% pentobarbital sodium (Merck, Rahway, NJ, USA), and blood samples were collected from the abdominal aorta. Serum was separated by centrifugation for subsequent assays. Gastrocnemius muscle samples were divided into three parts: one snap-frozen in liquid nitrogen and stored at −80 °C for further analysis, one preserved in electrolyte solution at −4 °C, and one fixed in 10% paraformaldehyde (Sangon Biotech, Shanghai, China) for histological examination.

### Outcome measures and methods

#### Weekly measurement of blood glucose and body weight

After each week of intervention, record the body weight and RBG levels of all rats. Each measurement was performed three times per rat, and the average value was used for analysis.

#### Measurement of serum TG, LDL-C, C-P, and FINS levels, and calculation of HOMA-IR and ISI

Serum triglyceride (TG) and low-density lipoprotein cholesterol (LDL-C) levels were determined using a biochemical analyzer (Shenzhen Myriad Biomedical Electronics Co., Ltd., model BS-240VET). C-peptide (C-P) and fasting insulin (FINS) levels were measured using ELISA kits (ZC-36086 and ZC-54525). All measurements were performed in triplicate, and the mean values were used for subsequent analysis. The homeostatic model assessment of insulin resistance (HOMA-IR) and insulin sensitivity index (ISI) were calculated as follows:

$$\text{HOMA-IR} = (\text{FBG} \times \text{FINS})/22.5$$

$$\text{ISI} = \text{ln}[1/(\text{FBG} \times \text{FINS})]$$

All assays were performed following the manufacturer’s instructions [60].

#### Histopathological examination of skeletal muscle using hematoxylin-eosin staining

Eight gastrocnemius muscle tissue samples (1 mg each) from each group were fixed in 4% paraformaldehyde for 24 h. Samples were dehydrated using an automated dehydrator through sequential ethanol and xylene washes (Sinopharm Chemical Reagent Co., Ltd., Shanghai, China), then embedded in paraffin. Cross-sections were cut using a microtome (Leica RM2016, Leica, Germany); mounted on slides and oven-dried for 2 h; then deparaffinized, stained with hematoxylin (Seville Biotechnology Co., Ltd., Wuhan, China) and eosin (Bomei Biotechnology Co., Ltd., Hefei, China), dehydrated, air-dried, and sealed with neutral gum. Morphological features were examined microscopically. Eight rats per group were examined, with three non-overlapping fields randomly selected per tissue; the most representative H&E images were presented. Muscle tissues were examined, with three non-overlapping fields randomly selected per tissue; the most representative H&E images were presented.

#### ATP measurement in skeletal muscle tissue using a commercial assay kit

Eight gastrocnemius muscle tissues per group were analyzed, with ATP levels measured three times for each sample and the results averaged. Twenty milligrams of fresh gastrocnemius muscle tissue were lysed on ice. Standards and working solutions were prepared on ice. ATP content was measured by recording relative light units (RLU) using a luminometer. Procedures strictly followed the manufacturer’s instructions.

#### Western blot analysis of AMPK, PGC-1α, TFAM, and GLUT4 proteins in skeletal muscle tissue

Two hundred milligrams of gastrocnemius muscle tissue were homogenized in 2 mL lysis buffer (1:10 w/v) using a high-speed, low-temperature tissue grinder (−20 °C, four cycles of 60 s). The homogenate was incubated on ice for 30 min, then centrifuged at 12,000 rpm and 4 °C for 10 min. Protein concentration of the supernatant was measured using a BCA assay (REF: P0009, Beyotime Biotechnology, Shanghai, China). Proteins were separated by electrophoresis on Precast Protein Plus Gels and transferred to PVDF membranes (REF: ISEQ00010, Sigma-Aldrich, St. Louis, MO, USA), which were rinsed with PBS and blocked with 5% skim milk in TBST. Membranes were incubated with primary antibodies (β-actin (REF: AC026, Abclonal, Wuhan, China), AMPK (REF: GB112669, Servicebio, Wuhan, China), GLUT4 (REF: 347063, Zen-Bio, Chengdu, China), PGC-1α (REF: 381615, Zen-Bio, Chengdu, China), and TFAM (REF: AF0531, Affbiotech, Beijing, China)) overnight at 4 °C, washed, then incubated with secondary antibodies (dilution: 1:5000) at room temperature. After final washing, ECL imaging was performed, and bands were analyzed using Tanon Fluorescence Image Analysis System V2.0. Protein expression levels were normalized to β-actin. Each parameter was measured in triplicate for 8 rats per group and averaged. Primary antibodies used for Western blot were as follows: AMPK (1:1000); PGC-1α (1:1000); GLUT4(1:1000); TFAM(1:2000); β-actin (1:50000).

#### Quantification of AMPK, PGC-1α, TFAM, and GLUT4 gene expression in skeletal muscle tissue by RT-PCR

Twenty milligrams of frozen gastrocnemius muscle tissue were homogenized and centrifuged; total RNA was extracted using standard protocols. RNA concentration and purity were assessed by spectrophotometry, followed by genomic DNA removal. Reverse transcription was performed per kit instructions (REF: 19221ES50, YEASEN, Shanghai, China) in a 20 µL reaction system (42 °C for 2 min, 37 °C for 15 min, 85 °C for 5 s). cDNA was stored at − 80 °C. PCR amplification was conducted with initial denaturation at 95 °C for 30 s, followed by 45 cycles of 95 °C for 5 s, 55 °C for 30 s, and 72 °C for 30 s. Fluorescence signals were recorded during the final extension of each cycle. Relative mRNA levels were calculated using the 2^-ΔΔCT method, with β-actin as internal reference. Primers were designed and synthesized by Shanghai Sangong Bioengineering Technology Service Co. Each parameter was measured in triplicate and averaged.

### Statistical analysis

All experimental data were analyzed using SPSS 27.0 statistical software (IBM, Armonk, NY, USA). Data conforming to normal distribution are presented as mean ± standard deviation ($\:\stackrel{\text{-}}{\text{X}}\pm\text{S}$). One-way ANOVA was performed when data met normality and homogeneity of variance assumptions; pairwise comparisons were conducted using the LSD test. Repeated measures ANOVA was used to analyze body weight and RBG over time. When homogeneity of variance was not met, the nonparametric Kruskal-Wallis test was applied. Statistical graphs were generated using GraphPad Prism 9.0. The sequences of primers and bases are listed in Table 1.

**Table 1:** Primer sequences for RT-PCR

Target genes		Primer sequences	Product size/bp
β-actin	Forward	GGAAATCGTGCGTGACATT	76
	Reverse	GCGGCAGTGGCCATCTC	
AMPK	Forward	CCCACAGAAATCCAAACACCAAGG	118
	Reverse	GTCCAACTGCTTGATTGCTCTACAC	
GLUT4	Forward	TCCTCCTGCTTGGCTTCTTCATC	127
	Reverse	CTGGGTTTCACCTCCTGCTTAAG	
PGC-1α	Forward	AGGAAGATGAGGAGGAGGAAGAGG	146
	Reverse	CCGCACAGGGCACACAGAG	
TFAM	Forward	GGGAATGTGGGGCGTGCTAAG	88
	Reverse	GCTGACAGGCGAGGGTATGC	

## Result

### EA reduces body weight fluctuation in T2DM model rats

**Fig. 2:**
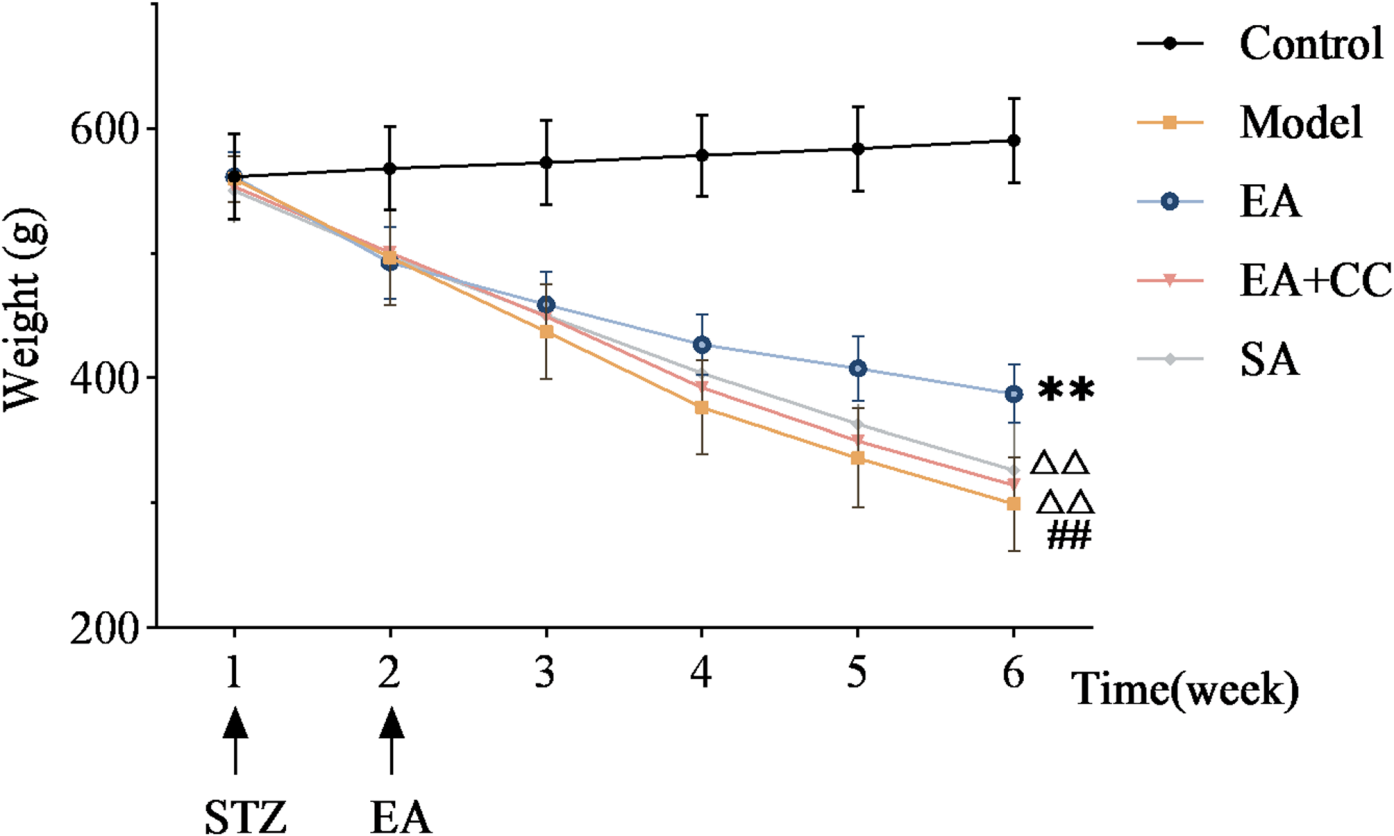
Weekly fluctuations in body weight of rats before and after modeling and electroacupuncture (EA) intervention (*n* = 8 per group). “1 week” denotes the time point following 8 weeks of high-fat, high-sugar diet prior to STZ intraperitoneal injection; “2 week” marks 1 week after STZ injection and the start of EA intervention; “3–6 week” correspond to the end of weeks1-4 of EA treatment, respectively. EA: electroacupuncture group; EA + CC: EA combined with the AMPK inhibitor Compound C; SA: sham acupuncture group. #*P* < 0.05, ##*P* < 0.01 vs. control group; *P < 0.05, **P < 0.01 vs. model group; △*P* < 0.05, △△*P* < 0.01 vs. EA group

Figure 2 presents line charts illustrating weekly body weight changes in each group of rats after intervention. Before modeling, body weight of rats in the model, EA, EA + CC, and SA groups were significantly higher than those in the control group (*P* < 0.05), indicating that high-sugar and high-fat feeding increased body weight. Following high-sugar and high-fat feeding combined with intraperitoneal STZ injection, rats in the model group exhibited a rapid decline in body weight that persisted for one week. After one week of EA intervention, no significant differences in body weight were observed among the EA, EA + CC, SA, and model groups (*P* > 0.05). Body weight trends during the second week of EA intervention remained consistent with those in the first week. After three weeks of EA intervention, the EA group exhibited significantly higher body weight than the model group (*P* < 0.05, η² =0.925), whereas the EA + CC and SA groups showed no significant changes (*P* > 0.05). At the fourth week, body weight changes were similar to those at week three. The EA group exhibited a slower rate of weight loss compared to the model group (*P* < 0.01, η² =0.934), while the EA + CC and SA groups showed no significant changes (*P* > 0.05). Compared to the EA group, the EA + CC and SA groups exhibited significantly higher weight (*P* < 0.01). These findings suggest that EA can alleviate T2DM-induced weight loss, and that the AMPK antagonist Compound C attenuates this therapeutic effect of EA.

### EA reduces RBG fluctuation range and FBG levels in T2DM model rats

**Fig. 3:**
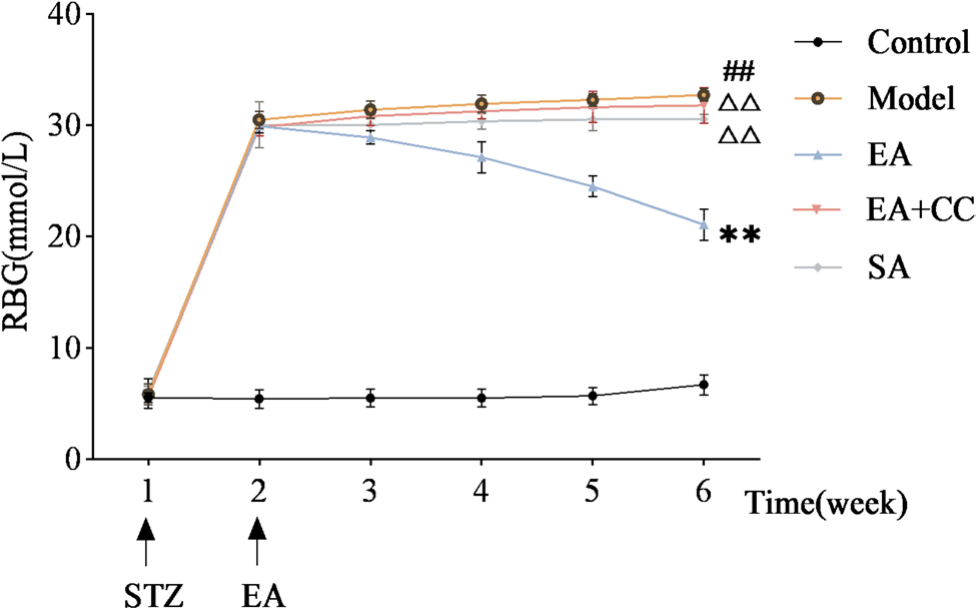
Weekly fluctuations in random blood glucose (RBG) levels of rats before and after modeling and intervention (*n* = 8 per group). “1 week” indicates the time point following 8 weeks of high-fat, high-sugar feeding prior to intraperitoneal STZ injection. “2 week” marks 1 week after STZ injection and the initiation of EA intervention. “3–6 week” represent the end of weeks 1–4 of EA treatment, respectively. EA: electroacupuncture group; EA + CC: EA combined with the AMPK inhibitor Compound C; SA: sham acupuncture group. #*P* < 0.05, ##*P* < 0.01 vs. control group; *P < 0.05, **P < 0.01 vs. model group; △*P* < 0.05, △△*P* < 0.01 vs. EA group

Figure 3 illustrates the weekly RBG fluctuations in each group of rats. Following intraperitoneal STZ injection combined with high-sugar and high-fat feeding, RBG rapidly increased and remained above 16.7 mmol/L for one week, with no significant differences among the four groups (*P* > 0.05), confirming successful and stable establishment of the T2DM model. During the first week of EA intervention, no significant differences in RBG were observed among the EA, EA + CC, SA, and model groups (all *P* > 0.05). In the second week, the EA group exhibited a significant reduction in RBG compared to the model group (*P* < 0.01), whereas the EA + CC and SA groups showed no significant changes (*P* > 0.05). The third week showed a consistent trend with the second week. At the fourth week, the EA group demonstrated a further significant decrease in RBG compared to the previous week (*P* < 0.01, η² =0.99), while the EA + CC and SA groups remained unchanged (*P* > 0.05).

**Fig. 4:**
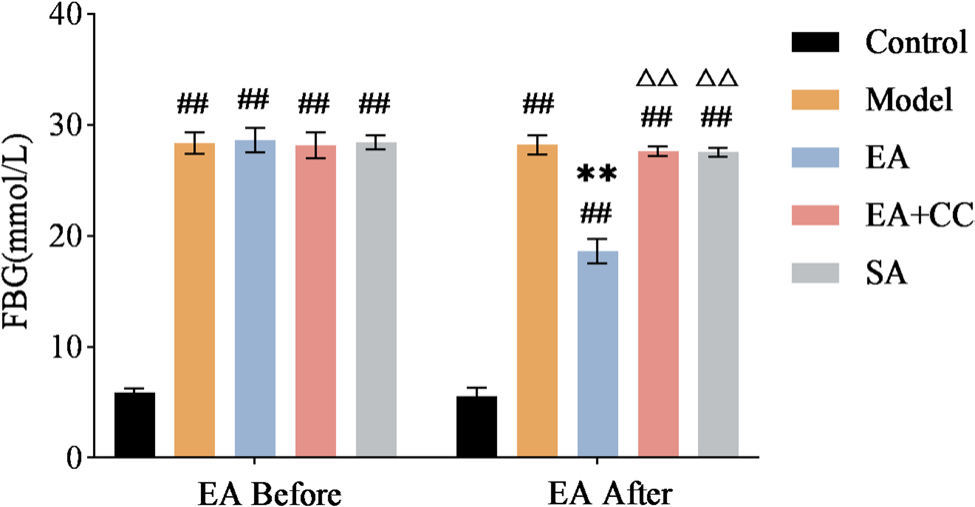
Changes in fasting blood glucose (FBG) levels in each group of rats before and after EA intervention (*n* = 8 per group). “Before” represents the time point after successful T2DM modeling and prior to EA treatment; “After” indicates the time point following 4 weeks of EA intervention. EA: electroacupuncture group; EA + CC: EA combined with the AMPK inhibitor Compound C; SA: sham acupuncture group. #*P* < 0.05, ##*P* < 0.01 vs. control group; *P < 0.05, **P < 0.01 vs. model group; △*P* < 0.05, △△*P* < 0.01 vs. EA group

Figure 4 presents changes in FBG levels in each group of rats before and after EA intervention. Compared to the control group, the model group exhibited significantly elevated FBG (*P* < 0.01). Compared to the model group, the EA group showed a significant reduction in FBG (*P* < 0.01), while the EA + CC and SA groups showed no significant changes (*P* > 0.05, η² =0.993). These results indicate that EA significantly reduces RBG and FBG in model rats. Compared to the EA group, the EA + CC and SA groups exhibited significantly higher FBG (*P* < 0.01), suggesting that Compound C inhibits the hypoglycemic effect of EA.

### EA reduces serum lipid levels in T2DM model rats

**Fig. 5:**
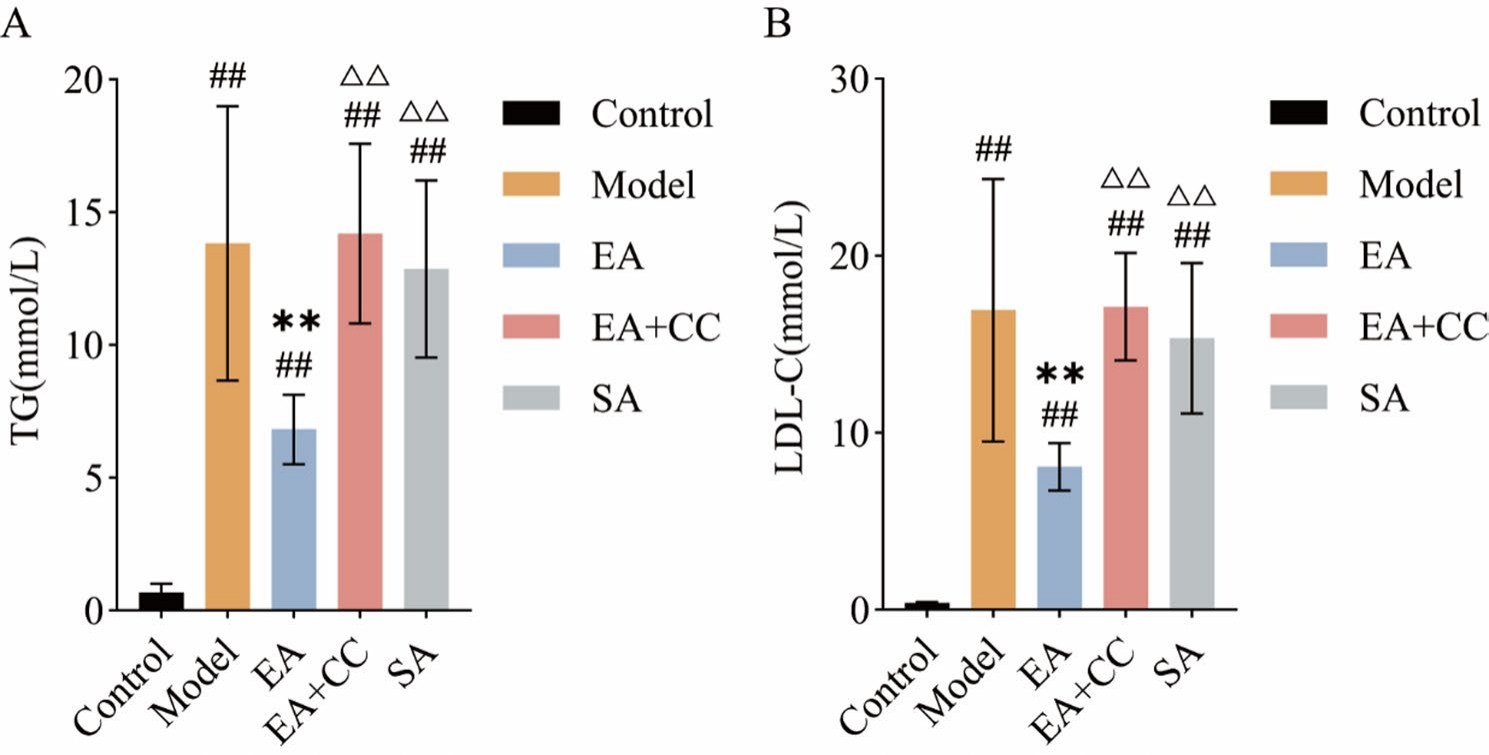
Comparison of serum triglyceride (TG) and low-density lipoprotein cholesterol (LDL-C) levels among groups after EA intervention (*n* = 8 per group). **A**: TG levels; **B**: LDL-C levels. EA: electroacupuncture group; EA + CC: EA combined with the AMPK inhibitor Compound C; SA: sham acupuncture group. #*P* < 0.05, ##*P* < 0.01 vs. control group; *P < 0.05, **P < 0.01 vs. model group; △*P* < 0.05, △△*P* < 0.01 vs. EA group

Figure 5 shows the serum TG and LDL-C levels in each group after four weeks of EA intervention. After four weeks of intervention, the model group showed significantly elevated serum TG and LDL-C levels compared to the control group (*P* < 0.01). Compared to the model group, the EA group exhibited significantly reduced serum TG and LDL-C levels (*P* < 0.01, LDL-C: η² =0.742; TG: η² =0.754), while no significant changes were observed in the EA + CC and SA groups (*P* > 0.05). Compared to the EA group, the EA + CC and SA groups exhibited significantly higher TG and LDL-C levels (*P* < 0.01). These findings indicate that EA effectively reduces serum lipid levels in T2DM rats.

### EA reduces serum insulin levels, improving hyperinsulinemia and insulin resistance

**Fig. 6:**
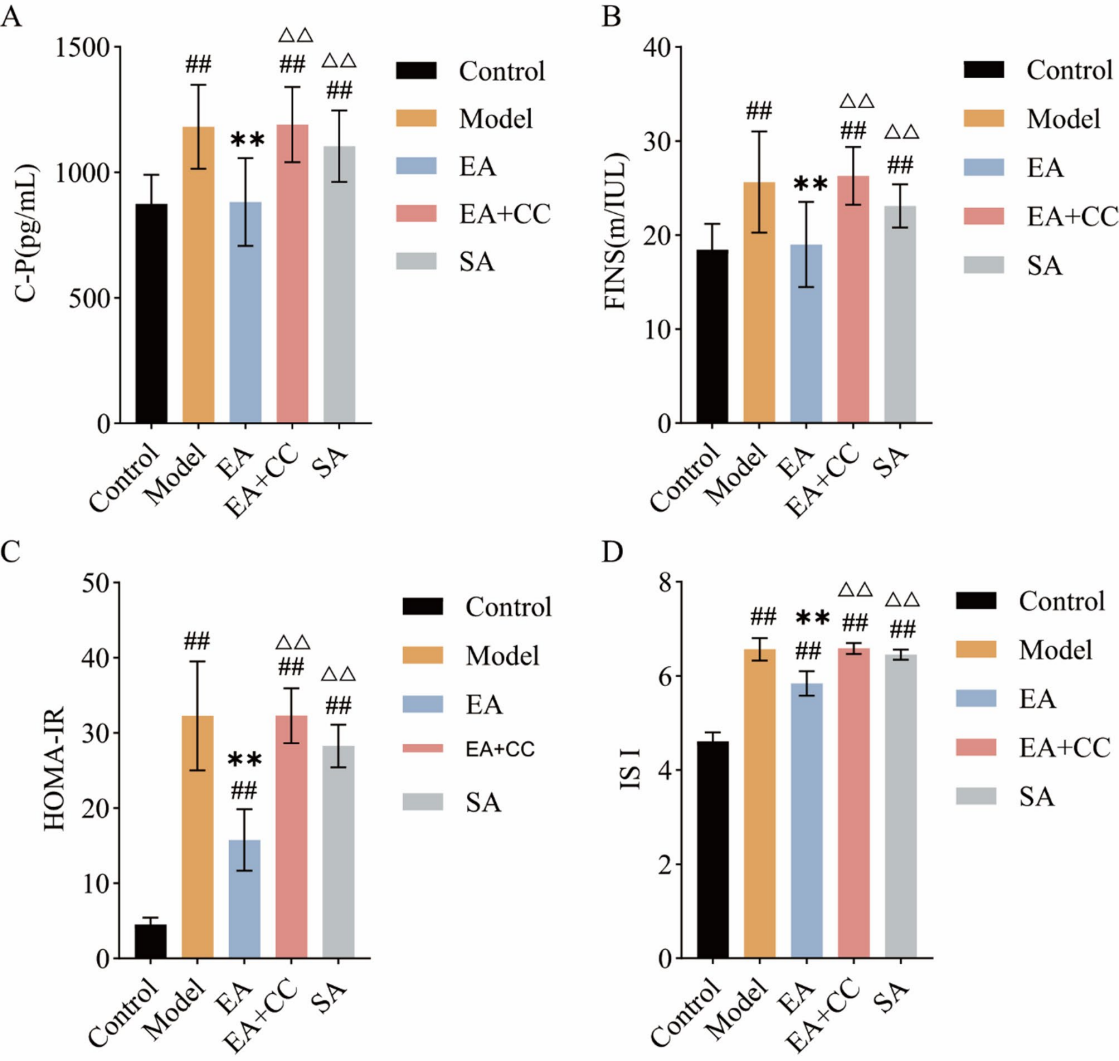
Comparison of C-peptide (C-P), fasting insulin (FINS), HOMA-IR, and the absolute value of insulin sensitivity index (ISI) among groups after EA intervention (*n* = 8 per group). **A**: C-P levels; **B**: FINS levels; **C**: HOMA-IR levels; **D**: Absolute value of ISI. Since ISI values are negative, higher absolute values indicate lower insulin sensitivity, while lower absolute values indicate higher sensitivity. EA: electroacupuncture group; EA + CC: EA combined with the AMPK inhibitor Compound C; SA: sham acupuncture group. #*P* < 0.05, ##*P* < 0.01 vs. control group; *P < 0.05, **P < 0.01 vs. model group; △*P* < 0.05, △△*P* < 0.01 vs. EA group

Figure 6 illustrates the differences in serum FINS, C-P, HOMA-IR, and the absolute value of ISI levels among the groups. After 4 weeks of intervention, compared to the control group, the model group exhibited significantly elevated serum FINS, C-P, HOMA-IR and the absolute value of ISI levels (all *P* < 0.01). Compared to the model group, the EA group showed significantly decreased serum FINS, C-P, HOMA-IR and the absolute value of ISI levels(all *P* < 0.01, FINS: η² =0.459; C-P: η² =0.496; HOMA-IR: η² =0.881; ISI: η² =0.945). No significant changes in FINS, C-P, HOMA-IR, and the absolute value of ISI levels were observed in the EA + CC and SA groups compared to the model group (all *P* > 0.05). Compared to the EA group, the EA + CC and SA groups exhibited significantly increased serum FINS, C-P, HOMA-IR and the absolute value of ISI levels (all *P* < 0.01). These results indicate that EA can improve IRand hyperinsulinemia in T2DM rats, while Compound C reverses this beneficial effect of EA.

### EA increases skeletal muscle ATP content to repair muscle morphology and improve glucose metabolism disorders

**Fig. 7:**
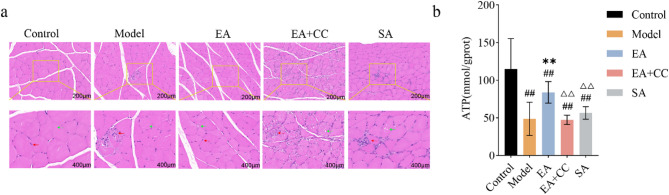
**A**: Representative histological images of the gastrocnemius muscle in each group following EA intervention (*n* = 8 per group). Scale bars: 200 μm (top row), 400 μm (bottom row). Red arrows indicate nuclei; green arrows indicate intracellular lipid droplets. EA: electroacupuncture group; EA + CC: EA combined with the AMPK inhibitor Compound C. **B**: Comparison of ATP content in gastrocnemius muscle tissue among groups after EA intervention (*n* = 8 per group). C: control group; M: T2DM model group; EA: electroacupuncture group; EA + CC: EA combined with the AMPK inhibitor Compound C; SA: sham acupuncture group. #*P* < 0.05, ##*P* < 0.01 vs. control group; *P < 0.05, **P < 0.01 vs. model group; △*P* < 0.05, △△*P* < 0.01 vs. EA group

Figure 7a illustrates the differences in gastrocnemius muscle morphology among the groups. At 200× magnification, H&E staining of the gastrocnemius muscle in the control group showed intact muscle cross-sections with clear cell morphology and boundaries. Blue-stained nuclei were distinct, muscle cells were separated by white connective tissue, exhibiting regular oval shapes and tight, orderly arrangement without pathological changes. Compared to the control group, the mondel group showed disrupted gastrocnemius muscle cell structure, unclear boundaries, widened intercellular spaces, nuclear aggregation, and intracellular vacuole-like lipid droplets.

Compared to the model group, the EA group exhibited improved pathological features: clearer cell boundaries, slightly more regular morphology, tighter fiber arrangement, reduced nuclear aggregation, and disappearance of vacuole-like lipid droplets. In contrast, the EA + CC group showed more disordered fiber arrangement, extremely blurred boundaries, irregular cell morphology, increased nuclear aggregation, and more vacuole-like lipid droplets compared to the model group. No obvious pathological improvement was observed in the SA group compared to the model group.

The ATP content in gastrocnemius muscle was significantly decreased in the model group compared to the control group (*P* < 0.01). Compared to the model group, the EA group showed a significant increase in ATP content (*P* < 0.01, η² =0.611), whereas no significant changes were observed in the EA + CC and SA groups (*P* > 0.05). Compared to the EA group, ATP levels in the EA + CC and SA groups were significantly reduced (*P* < 0.01) (Fig. 7b).

### EA promotes skeletal muscle glucose metabolism via activation of the AMPK/PGC-1α/TFAM signaling pathway

**Fig. 8:**
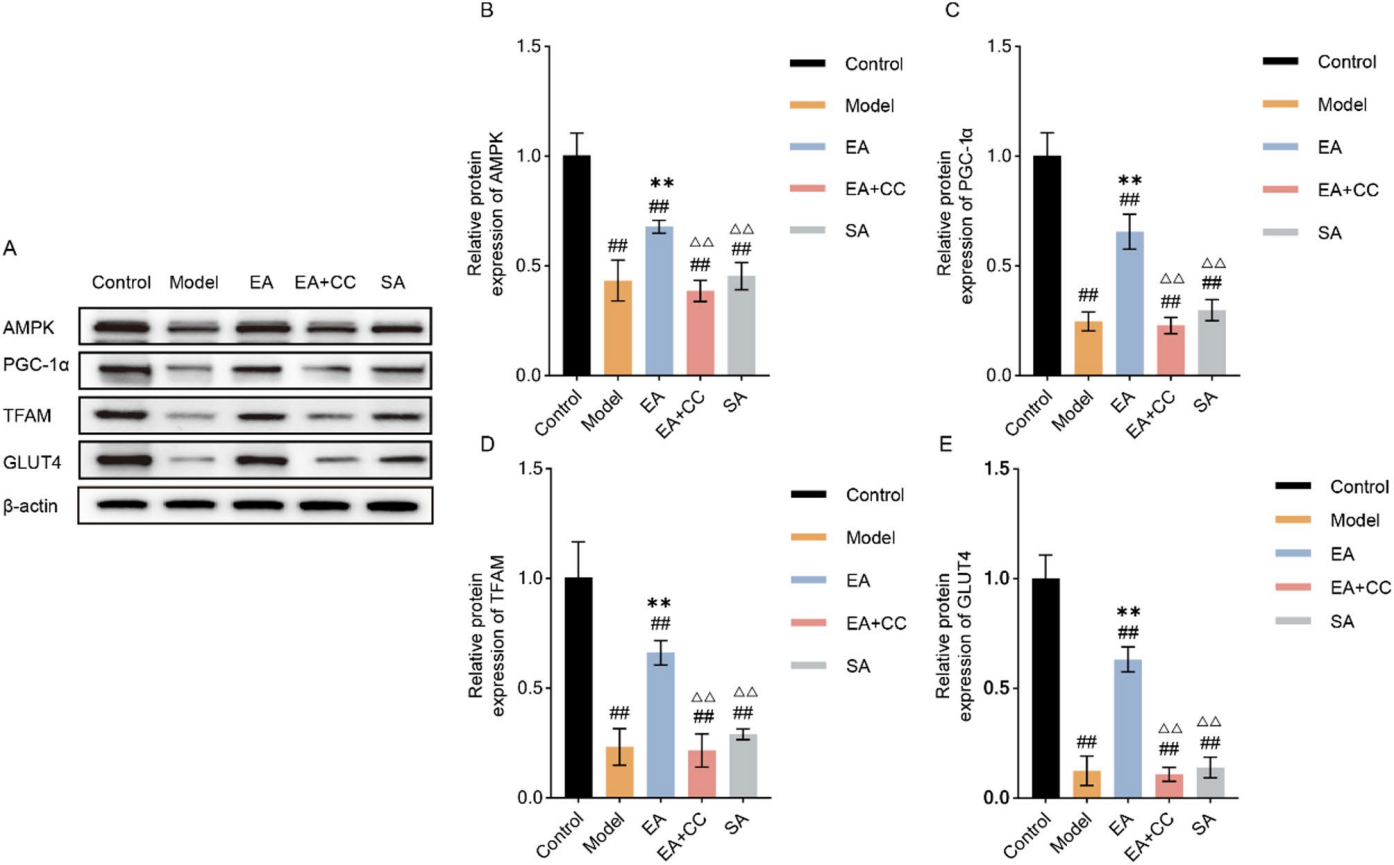
Protein expression levels of AMPK, PGC-1α, TFAM, and GLUT4 in gastrocnemius muscle tissue following EA intervention (*n* = 8 per group). **A**: Representative Western blot bands; **B**: Quantitative comparison of AMPK protein expression; **C**: PGC-1α protein expression; **D**: TFAM protein expression; **E**: GLUT4 protein expression. EA: electroacupuncture group; EA + CC: EA combined with the AMPK inhibitor Compound C; SA: sham acupuncture group. #*P* < 0.05, ##*P* < 0.01 vs. control group; *P < 0.05, **P < 0.01 vs. model group; △*P* < 0.05, △△*P* < 0.01 vs. EA group

**Fig. 9:**
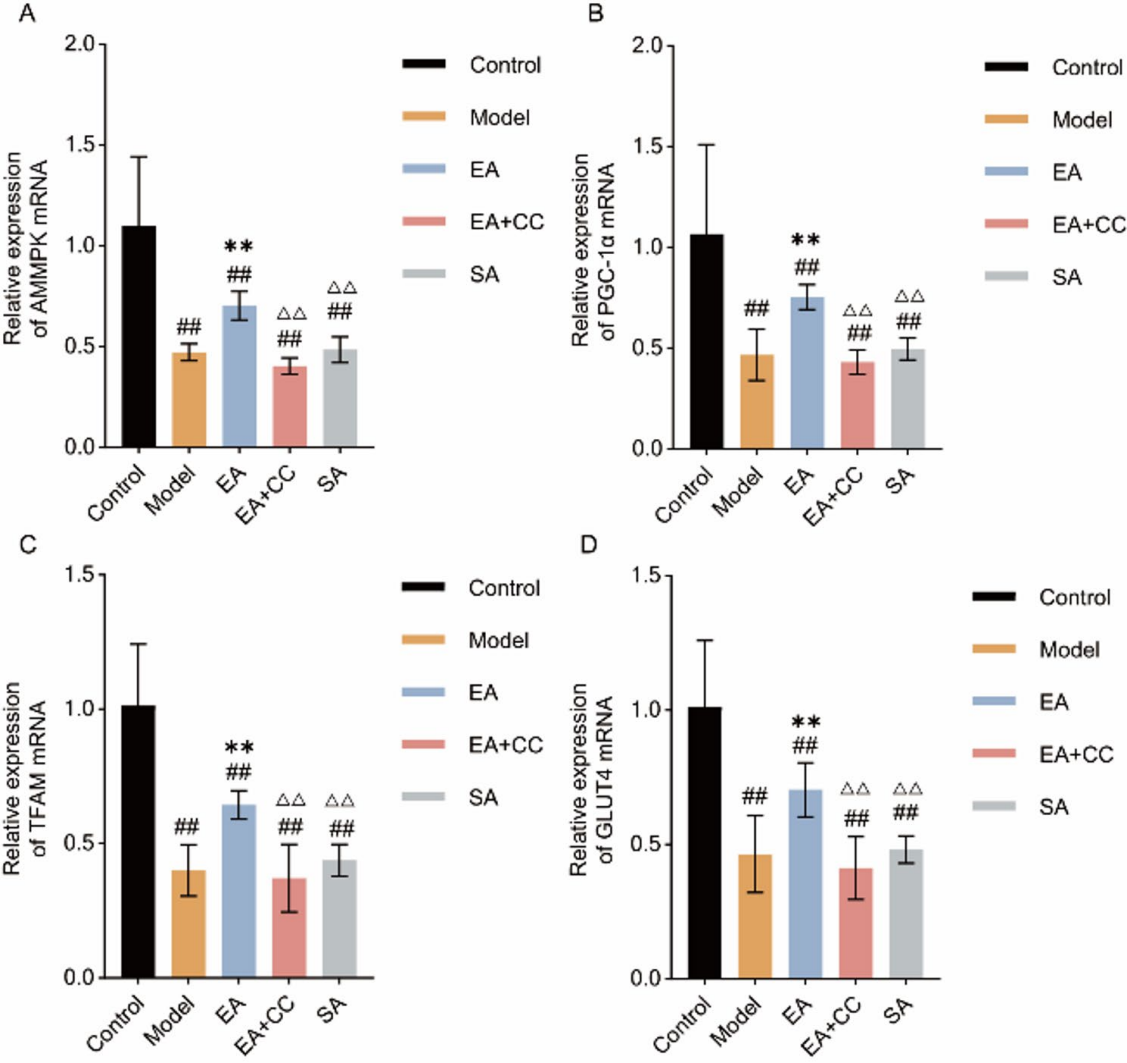
Relative mRNA expression levels of AMPK, PGC-1α, TFAM, and GLUT4 in gastrocnemius muscle tissue following EA intervention (*n* = 8 per group). **A**: AMPK mRNA expression; **B**: PGC-1α mRNA expression; **C**: TFAM mRNA expression; **D**: GLUT4 mRNA expression. EA: electroacupuncture group; EA + CC: EA combined with the AMPK inhibitor Compound C; SA: sham acupuncture group #*P* < 0.05, ##*P* < 0.01 vs. control group; *P < 0.05, **P < 0.01 vs. model group; △*P* < 0.05, △△*P* < 0.01 vs. EA group

Western blot and RT-PCR results are shown in Figs. 8 and 9. Compared to the control group, the model group exhibited significantly decreased protein and mRNA levels of AMPK, PGC-1α, TFAM, and GLUT4 in gastrocnemius muscle cells (all *P* < 0.01, AMPK: η² =0.921; PGC-1α: η² =0.944; TFAM:η² =0.908; GLUT4: η² =0.942. AMPK mRNA: η² =0.738; PGC-1α mRNA: η² =0.597; TFAM mRNA:η² =0.785; GLUT4 mRNA: η² =0.721). Compared to the model group, the EA group showed significant increases in both protein and gene expression levels of AMPK, PGC-1α, TFAM, and GLUT4 (all *P* < 0.01). No significant changes were observed in these markers in the EA + CC and SA groups compared to the model group (all *P* > 0.05). Compared to the EA group, the EA + CC and SA groups showed significantly reduced protein and gene expression levels of AMPK, PGC-1α, TFAM, and GLUT4 (all *P* < 0.01).

## Disscussion

T2DM is a chronic metabolic disorder that poses a significant threat to human health and well-being [10]. Clinically, it is characterized by polyphagia, polydipsia, polyuria, and weight loss [6]. In addition, T2DM is marked by hyperglycemia, dyslipidemia, hyperinsulinemia, and IR, and is frequently accompanied by complications involving microvascular damage to the retina and kidneys, as well as macrovascular lesions in the heart and brain [34]. In this study, after 8 weeks of high-sugar and high-fat feeding combined with low-dose intraperitoneal injection of STZ, model rats exhibited consistently elevated RBG levels (>16.7 mmol/L) for 7 consecutive days, along with rapid weight loss, indicating the successful and stable establishment of a T2DM animal model. T2DM is primarily characterized by glucose metabolic dysfunction, often accompanied by lipid abnormalities [11]. TG, the main form of energy storage in the body, when elevated in serum, can promote IR and impair glucose metabolism [51]. LDL-C can exacerbate diabetes progression [1]. Compared with the control group, significantly higher levels of RBG, FBG, TG, and LDL-C were observed in the model group, indicating severe glucolipid metabolic dysfunction. Following four weeks of EA intervention, these parameters were significantly reduced, suggesting an improvement in glucose and lipid metabolism. This finding is consistent with previous animal studies of acupuncture for T2DM using similar experimental designs, in which TG, TC, and LDL-C were reduced while HDL-C was elevated. These results further support the representativeness of the lipid indicators selected in this study [44, 59]. This effect was partially reversed by the AMPK antagonist, implying the involvement of AMPK signaling. Furthermore, EA attenuated the rapid weight loss observed in diabetic rats, indicating a potential protective effect against T2DM-associated weight reduction.

IR is a key mechanism in T2DM pathogenesis, impairing insulin signaling and GLUT4-mediated glucose uptake in peripheral tissues [59]. In IR conditions, increased serine phosphorylation of insulin receptor substrate-1(IRS-1) interferes with its interaction with phosphoinositide 3-kinase(PI3K), leading to decreased protein kinase B(PKB) phosphorylation and reduced translocation of GLUT4 to the cell membrane [22]. Clinical studies report a 40–60% reduction in skeletal muscle membrane GLUT4 density in T2DM patients, negatively correlating with fasting glucose and HOMA-IR [25]. These findings were consistent with our study, in which T2DM model rats exhibited elevated levels of FINS, C-P, HOMA-IR, and the absolute value of the ISI, indicating the presence of hyperinsulinemia and IR. EA intervention significantly reduced FINS, C-P, HOMA-IR, and absolute ISI levels, suggesting its efficacy in ameliorating hyperinsulinemia and IR, in line with previous clinical research. These effects were attenuated following administration of the AMPK antagonist Compound C.

Skeletal muscle is the largest insulin-sensitive tissue in the body, playing a central role in glucose metabolism and relying heavily on ATP produced via mitochondrial oxidative phosphorylation [27]. Previous studies have shown decreased ATP levels and morphological abnormalities in the skeletal muscles of patients with IR and T2DM [3, 46]. Given its high mitochondrial content, impaired oxidative phosphorylation in skeletal muscle reduces ATP production, directly inhibiting AMPK activity and diminishing its regulation of GLUT4 translocation [58]. Moreover, ATP deficiency forces skeletal muscle to rely on anaerobic glycolysis, leading to intracellular lactate accumulation and inhibition of glycogen synthase activity, thereby further reducing glucose storage [38]. Animal studies have confirmed that ATP levels, GLUT4 membrane localization, and glycogen content are reduced in the skeletal muscles of STZ-induced aged T2DM rats, and that these abnormalities can be significantly improved by metformin, thereby alleviating IR and enhancing glucose metabolism [24]. In this study, disrupted muscle fiber arrangement, cellular morphological damage, and significantly reduced ATP levels were observed in the skeletal muscles of T2DM rats induced by a high-sugar and high-fat diet combined with STZ, consistent with previous animal findings. EA was found to repair muscle cell and fiber damage and significantly increase ATP levels, whereas these effects were suppressed by the administration of an AMPK antagonist.

Skeletal muscle glucose metabolism is regulated by the AMPK/PGC-1α/TFAM signaling pathway, which is activated in an ATP-dependent manner [29]. Reduced ATP production has been shown in T2DM animal models to inhibit this pathway, leading to downregulation of nuclear-encoded mitochondrial genes, impaired mitochondrial biogenesis, and subsequent IR and glucose metabolic disorders, thereby accelerating T2DM progression [47]. AMPK, a key regulator of energy homeostasis, is activated by the AMP/ATP ratio [36]. In vitro studies have shown that AMPK expression is decreased under high glucose conditions and is significantly reduced in T2DM patients [15, 52]. AMPK activates TFAM via PGC-1α. TFAM, a key factor in nucleo-mitochondrial signaling, facilitates mitochondrial DNA transcription and replication, thereby alleviating metabolic dysfunction, impaired biosynthesis, and mitochondrial deficits [19]. TFAM deficiency in vivo results in rapid mtDNA depletion, impaired insulin secretion, and reduced glucose tolerance [33]. Activation of the AMPK/PGC-1α pathway has been shown to reduce inflammation and upregulate GLUT4 expression, thereby improving skeletal muscle glucose metabolism in T2DM rats [2, 42]. Exercise training activates the AMPK/PGC-1α pathway, enhancing mitochondrial biogenesis and fatty acid oxidation, which increases GLUT4 membrane expression [30]. In the present study, EA significantly upregulated the expression of AMPK, PGC-1α, TFAM, and GLUT4 protein and mRNA in the gastrocnemius muscle, whereas no significant changes were observed in the EA + CC and SA groups.

Compound C is a classical AMPK antagonist widely used in research on metabolic disorders such as diabetes, obesity, cancer, and neurodegenerative diseases [55]. Compound C competitively binds to the ATP-binding site of AMPK, inhibiting phosphorylation at the Thr172 residue of its catalytic α-subunit, thereby blocking AMPK-mediated fatty acid oxidation (via ACC phosphorylation inhibition) and glucose uptake (via inhibition of GLUT4 translocation), disrupting cellular energy homeostasis and metabolic regulatory networks [13, 23, 28, 56]. Literature review [8] indicates that 20 mg/kg is the commonly used dose in rat experiments, and this dosage was therefore adopted in the present study. To investigate the mechanisms underlying EA intervention in T2DM, an EA combined with Compound C treatment group was established. Results demonstrated that Compound C attenuated the regulatory effects of EA on glucose and lipid metabolism and IR improvement, and inhibited the EA-induced upregulation of AMPK, PGC-1α, TFAM, and GLUT4 protein and gene expression. These findings further suggest that EA may exert its hypoglycemic effects via activation of the AMPK/PGC-1α/TFAM signaling pathway.

Previous animal studies have reported that Guo et al. found the hypoglycemic effect of EA to be approximately 56% that of metformin [17]; Xihui et al. [59]. demonstrated that Bo’s abdominal acupuncture achieved a 78% reduction in FBG and an 80% improvement in IR compared to metformin; Shen et al. [44]. further demonstrated that EA combined with metformin produced a hypoglycemic effect nearly twice that of metformin alone. However, this study focused on the mechanistic targets of EA and did not include a positive drug control group, nor did it directly measure metabolic outcomes such as glycogen content, glucose uptake, or lipid parameters like TC and HDL-C, which represents a limitation. Future research should address this by including standard antidiabetic drugs and assessing potential synergistic effects with EA. In addition, The AMPK/PGC-1α/TFAM pathway was effectively inhibited by Compound C, supporting the hypothesis that EA may exert its effects on skeletal muscle glucose metabolism in T2DM via this axis. Future studies should evaluate other regulatory factors within this signaling pathway, such as Sirtuin 1(SIRT1) and NAD⁺-dependent deacetylase, and nicotinamide adenine dinucleotide (NAD⁺), to fully elucidate the hypoglycemic mechanism of EA.

This study provides a robust theoretical basis for the application of EA as a treatment strategy for T2DM in clinical settings, demonstrating its promising therapeutic potential. Moreover, EA may be integrated with existing pharmacological therapies to enhance clinical outcomes, particularly in patients with poor glycemic control or those experiencing adverse drug reactions.

## Conclusion

In this study, EA was shown to restore skeletal muscle cellular morphology and function, enhance ATP production, ameliorate hyperinsulinemia and IR, reduce blood glucose and lipid levels, and ultimately alleviate glucose metabolism disorders in T2DM rats, likely through AMPK/PGC-1α/TFAM pathway-mediated mitochondrial biogenesis and glucose metabolism regulation.

## Supplementary Information

Supplementary Material 1.

## Data Availability

No datasets were generated or analysed during the current study.
