# Supplementary material for: Electroacupuncture ameliorates glycolipid metabolism disorder in skeletal muscle of type 2 diabetic rats via modulation of the AMPK/PGC-1α/TFAM signaling pathway
Source: Diabetol Metab Syndr. 2025 Dec 30;17:464. doi: 10.1186/s13098-025-01960-w (PMC12754946; doi:10.1186/s13098-025-01960-w)
Supplement: Supplementary file 1 — Supplementary Material 1. [file 13098_2025_1960_MOESM1_ESM.zip › table and figure/table and figure.docx]

Table 1 Primer Sequences for RT-PCR

| Target genes |  | Primer Sequences | Product Size /bp |
| --- | --- | --- | --- |
| β-actin | Forward | GGAAATCGTGCGTGACATT | 76 |
|  | Reverse | GCGGCAGTGGCCATCTC |  |
| AMPK | Forward | CCCACAGAAATCCAAACACCAAGG | 118 |
|  | Reverse | GTCCAACTGCTTGATTGCTCTACAC |  |
| GLUT4 | Forward | TCCTCCTGCTTGGCTTCTTCATC | 127 |
|  | Reverse | CTGGGTTTCACCTCCTGCTTAAG |  |
| PGC-1α | Forward | AGGAAGATGAGGAGGAGGAAGAGG | 146 |
|  | Reverse | CCGCACAGGGCACACAGAG |  |
| TFAM | Forward | GGGAATGTGGGGCGTGCTAAG | 88 |
|  | Reverse | GCTGACAGGCGAGGGTATGC |  |


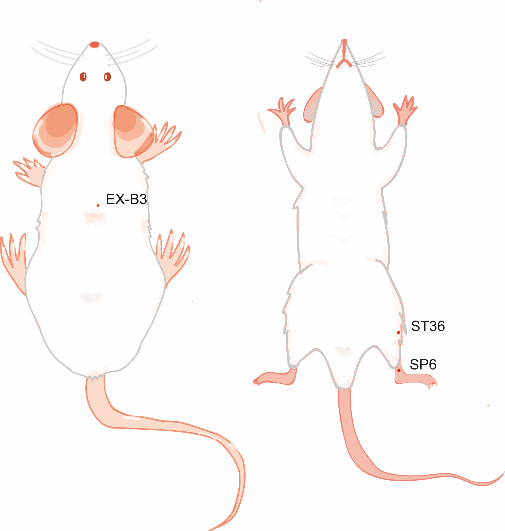


**Figure 1.** Localization of the acupoints ST36 (Zusanli), SP6 (Sanyinjiao), and EX-B3 (Weiwanxiashu) in rats. ST36, SP6, and EX-B3 represent the international codes for Zusanli, Sanyinjiao, and Weiwanxiashu, respectively.

**Figure 2.** Weekly fluctuations in body weight of rats before and after modeling and electroacupuncture (EA) intervention (n = 8 per group). “1 week” denotes the time point following 8 weeks of high-fat, high-sugar diet prior to STZ intraperitoneal injection; “2 week” marks 1 week after STZ injection and the start of EA intervention; “3-6 week” correspond to the end of weeks1-4 of EA treatment, respectively. EA: electroacupuncture group; EA+CC: EA combined with the AMPK inhibitor Compound C; SA: sham acupuncture group. #*P*< 0.05, ##*P*< 0.01 vs. control group; **P*< 0.05, ***P< 0.01 vs. model group; △P< 0.05, △△P< 0.01 vs. EA group.*

**Figure 3.** Weekly fluctuations in random blood glucose (RBG) levels of rats before and after modeling and intervention (n = 8 per group). “1 week” indicates the time point following 8 weeks of high-fat, high-sugar feeding prior to intraperitoneal STZ injection. “2 week” marks 1 week after STZ injection and the initiation of EA intervention. “3-6 week” represent the end of weeks 1–4 of EA treatment, respectively. EA: electroacupuncture group; EA+CC: EA combined with the AMPK inhibitor Compound C; SA: sham acupuncture group. #*P*< 0.05, ##*P*< 0.01 vs. control group; **P*< 0.05, ***P< 0.01 vs. model group; △P< 0.05, △△P< 0.01 vs. EA group.*

**Figure 4.** Changes in fasting blood glucose (FBG) levels in each group of rats before and after EA intervention (n = 8 per group). “Before” represents the time point after successful T2DM modeling and prior to EA treatment; “After” indicates the time point following 4 weeks of EA intervention. EA: electroacupuncture group; EA+CC: EA combined with the AMPK inhibitor Compound C; SA: sham acupuncture group. #*P*< 0.05, ##*P*< 0.01 vs. control group; **P*< 0.05, ***P< 0.01 vs. model group; △P< 0.05, △△P< 0.01 vs. EA group.*


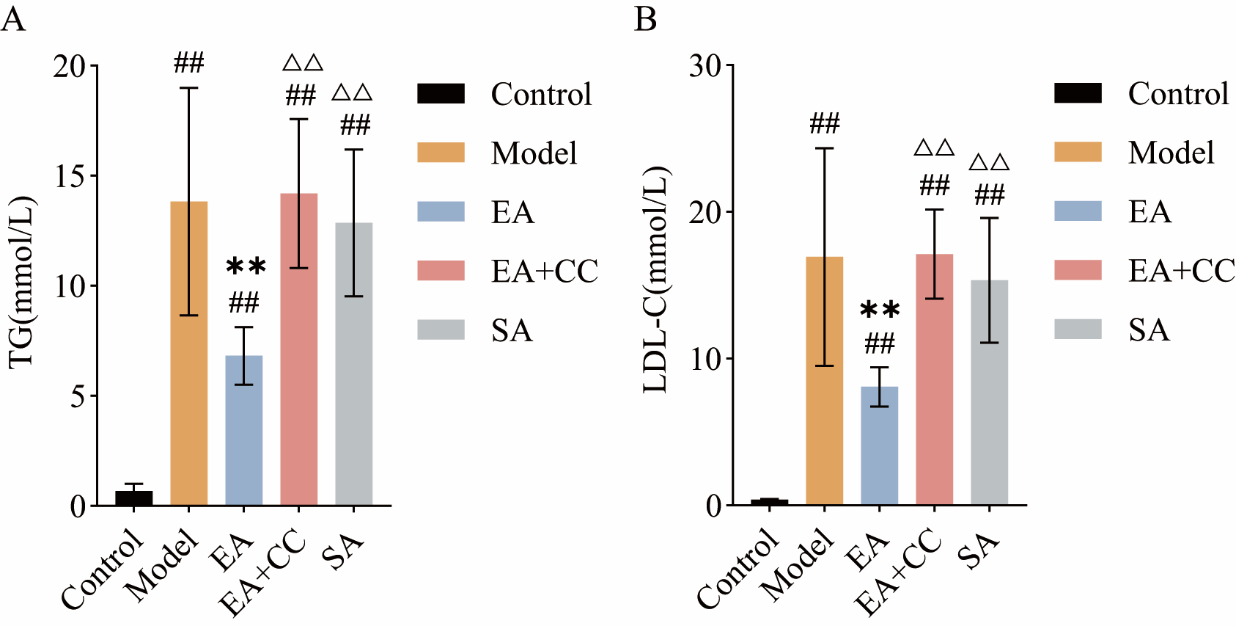


**Figure 5.** Comparison of serum triglyceride (TG) and low-density lipoprotein cholesterol (LDL-C) levels among groups after EA intervention (n = 8 per group). A: TG levels; B: LDL-C levels. EA: electroacupuncture group; EA+CC: EA combined with the AMPK inhibitor Compound C; SA: sham acupuncture group. #*P* < 0.05, ##*P* < 0.01 vs. control group; **P* < 0.05, ***P < 0.01 vs. model group; △P < 0.05, △△P < 0.01 vs. EA group.*


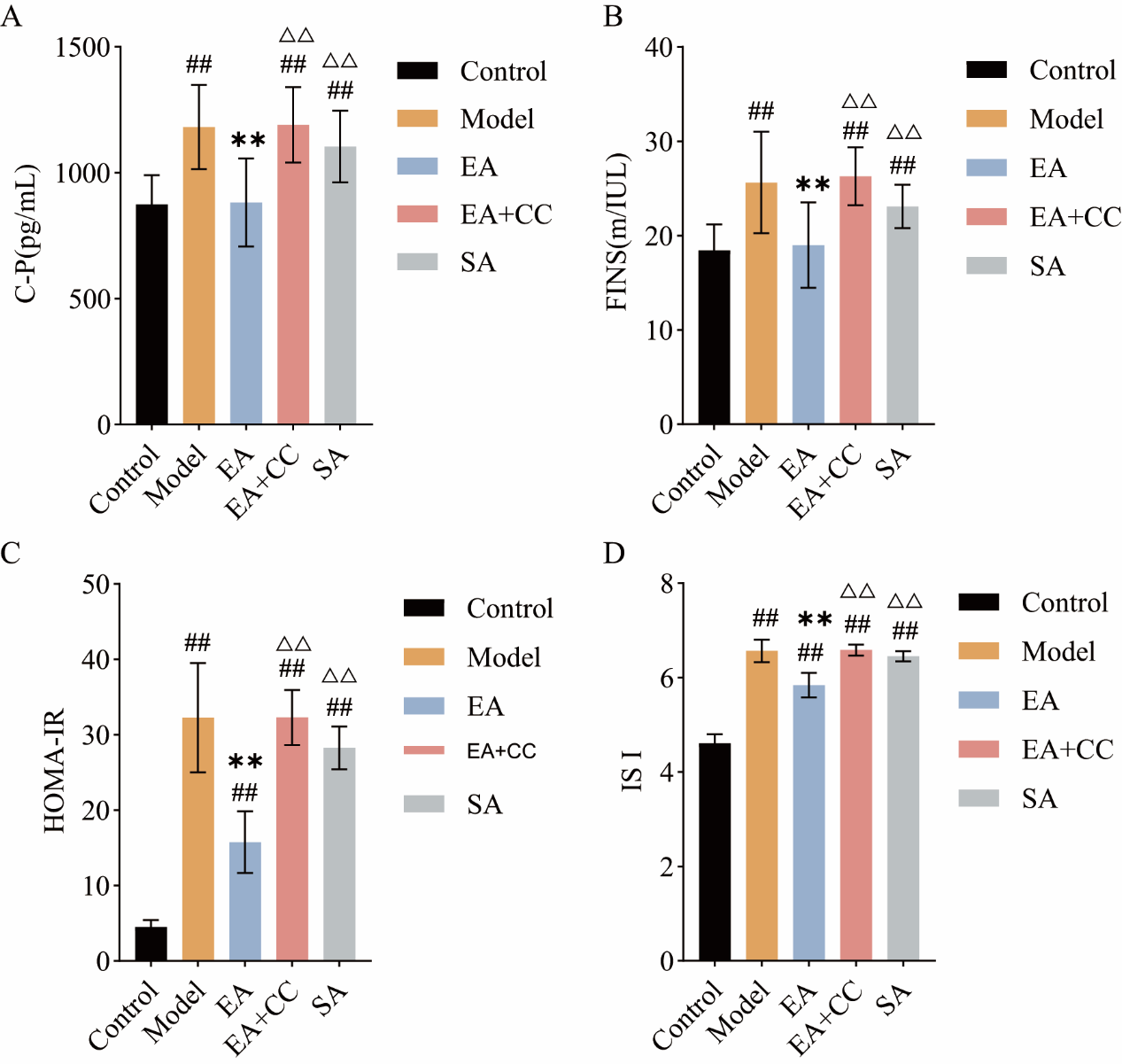


**Figure 6.** Comparison of C-peptide (C-P), fasting insulin (FINS), HOMA-IR, and the absolute value of insulin sensitivity index (ISI) among groups after EA intervention (n = 8 per group). A: C-P levels; B: FINS levels; C: HOMA-IR levels; D: Absolute value of ISI. Since ISI values are negative, higher absolute values indicate lower insulin sensitivity, while lower absolute values indicate higher sensitivity. EA: electroacupuncture group; EA+CC: EA combined with the AMPK inhibitor Compound C; SA: sham acupuncture group. #*P*< 0.05, ##*P*< 0.01 vs. control group; **P*< 0.05, ***P< 0.01 vs. model group; △P< 0.05, △△P< 0.01 vs. EA group.*


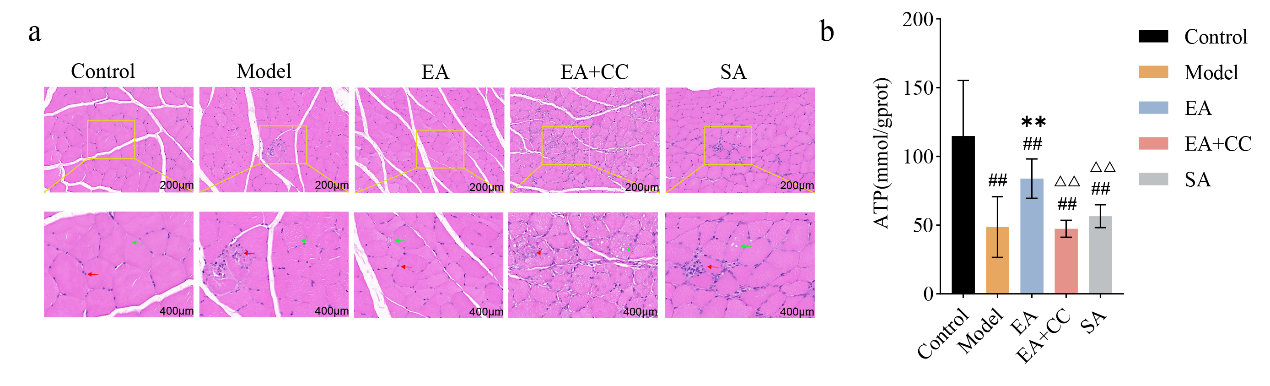


**Figure 7.** A: Representative histological images of the gastrocnemius muscle in each group following EA intervention (n = 8 per group). Scale bars: 200 μm (top row), 400 μm (bottom row). Red arrows indicate nuclei; green arrows indicate intracellular lipid droplets. EA: electroacupuncture group; EA+CC: EA combined with the AMPK inhibitor Compound C. B: Comparison of ATP content in gastrocnemius muscle tissue among groups after EA intervention (n = 8 per group). C: control group; M: T2DM model group; EA: electroacupuncture group; EA+CC: EA combined with the AMPK inhibitor Compound C; SA: sham acupuncture group. #*P*< 0.05, ##*P*< 0.01 vs. control group; **P*< 0.05, ***P< 0.01 vs. model group; △P< 0.05, △△P< 0.01 vs. EA group.*


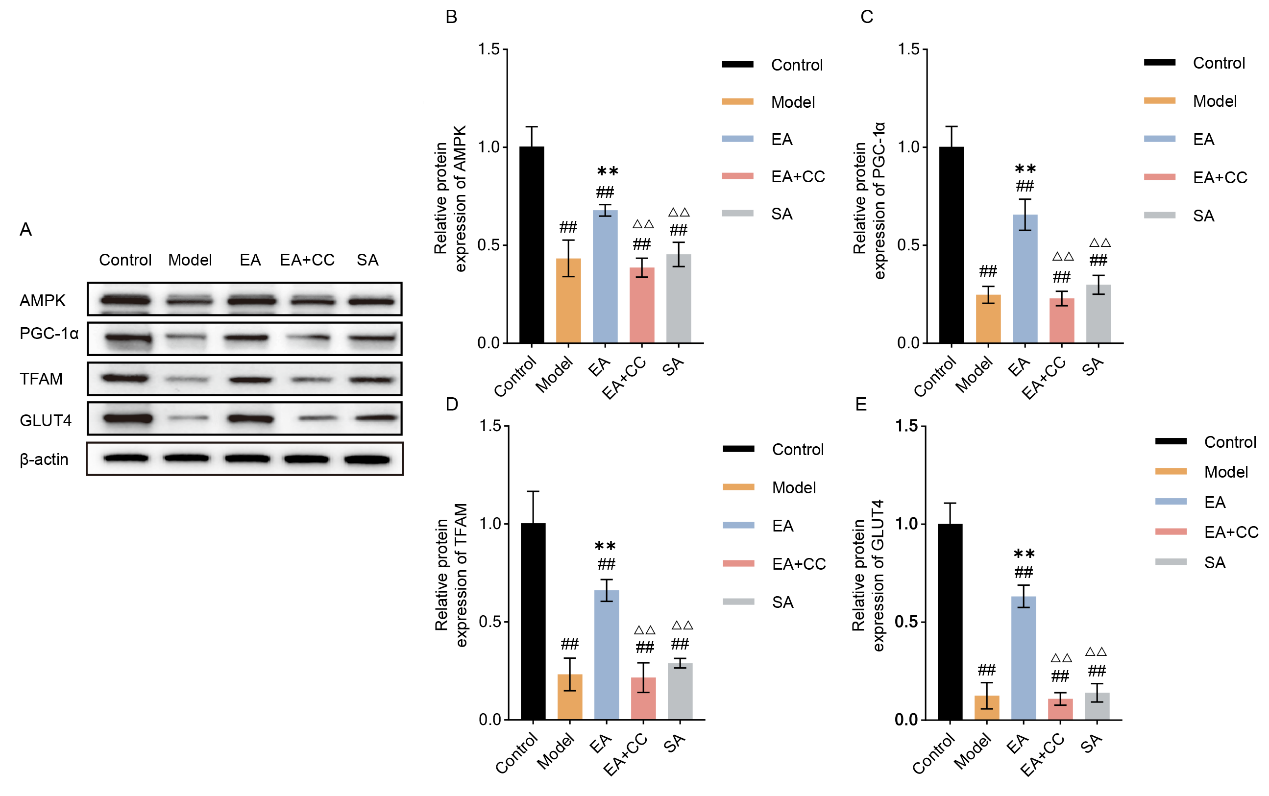


**Figure 8.** Protein expression levels of AMPK, PGC-1α, TFAM, and GLUT4 in gastrocnemius muscle tissue following EA intervention (n = 8 per group). **A:** Representative Western blot bands; **B:** Quantitative comparison of AMPK protein expression; **C:** PGC-1α protein expression; **D:** TFAM protein expression; **E:** GLUT4 protein expression. EA: electroacupuncture group; EA+CC: EA combined with the AMPK inhibitor Compound C; SA: sham acupuncture group. #*P*< 0.05, ##*P*< 0.01 vs. control group; **P*< 0.05, ***P< 0.01 vs. model group; △P< 0.05, △△P< 0.01 vs. EA group.*


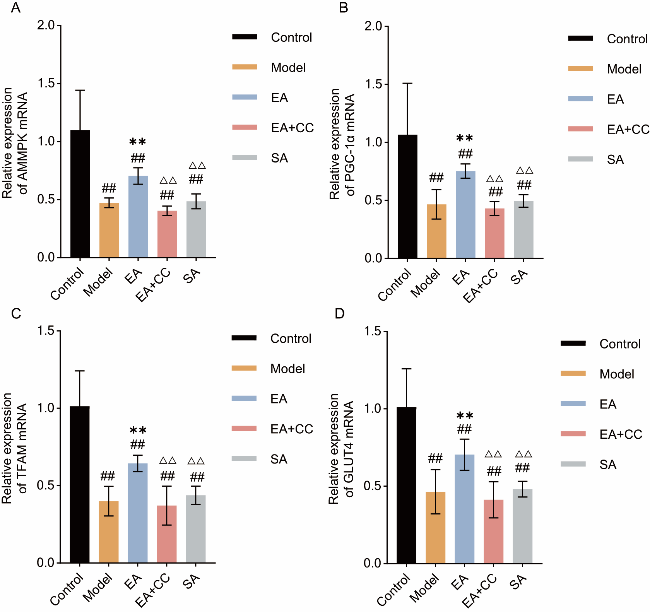


**Figure 9.** Relative mRNA expression levels of AMPK, PGC-1α, TFAM, and GLUT4 in gastrocnemius muscle tissue following EA intervention (n = 8 per group). **A:** AMPK mRNA expression; **B:** PGC-1α mRNA expression; **C:** TFAM mRNA expression; **D:** GLUT4 mRNA expression. EA: electroacupuncture group; EA+CC: EA combined with the AMPK inhibitor Compound C; SA: sham acupuncture group.
#*P*< 0.05, ##*P*< 0.01 vs. control group; **P*< 0.05, ***P< 0.01 vs. model group; △P< 0.05, △△P< 0.01 vs. EA group.*
