# Supplementary figures and images for: Electroacupuncture ameliorates glycolipid metabolism disorder in skeletal muscle of type 2 diabetic rats via modulation of the AMPK/PGC-1α/TFAM signaling pathway
Source: Diabetol Metab Syndr. 2025 Dec 30;17:464. doi: 10.1186/s13098-025-01960-w (PMC12754946; doi:10.1186/s13098-025-01960-w)

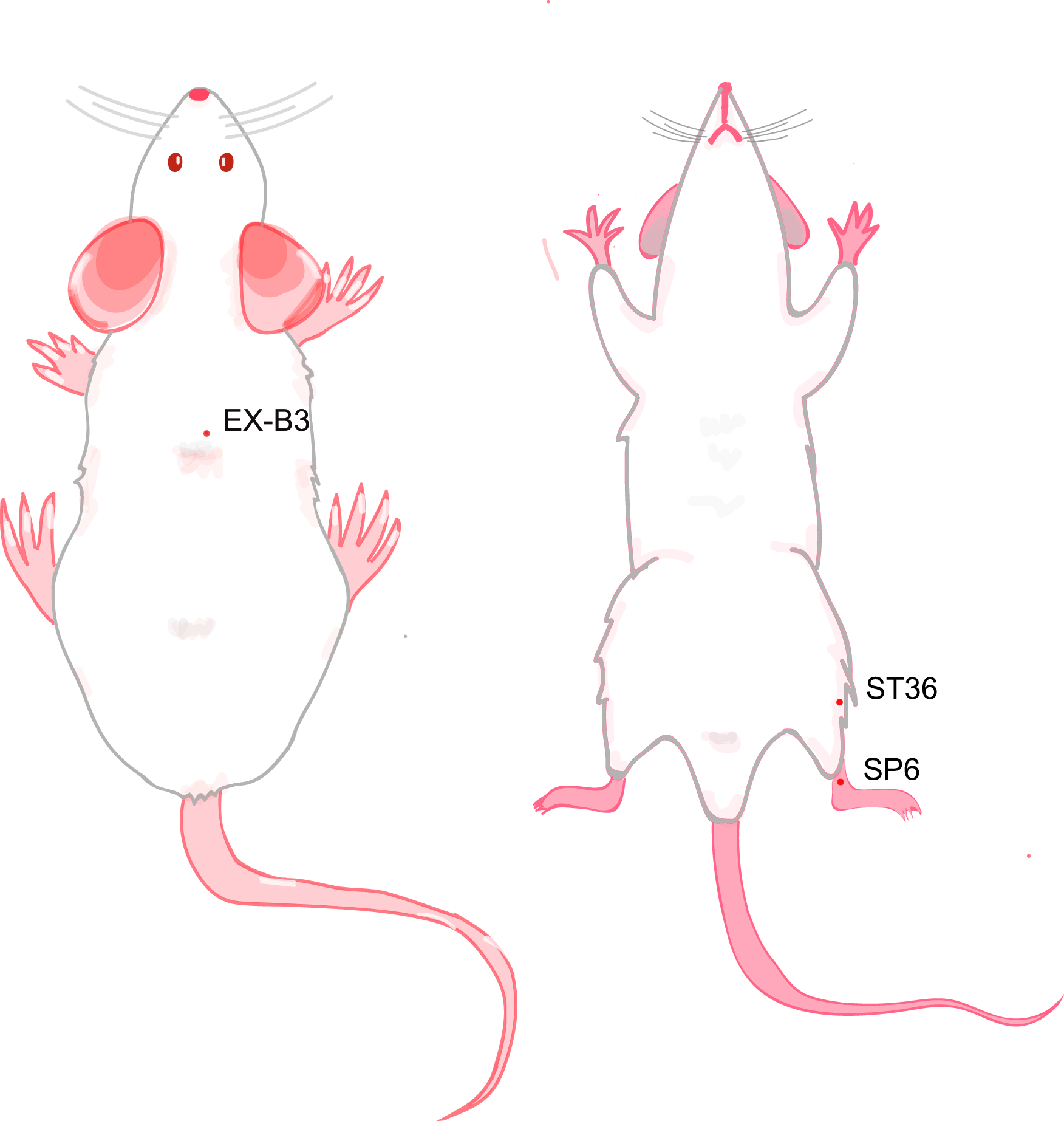

Supplement: Supplementary file 1 — Supplementary Material 1. [file 13098_2025_1960_MOESM1_ESM.zip › table and figure/acupoint.tif]

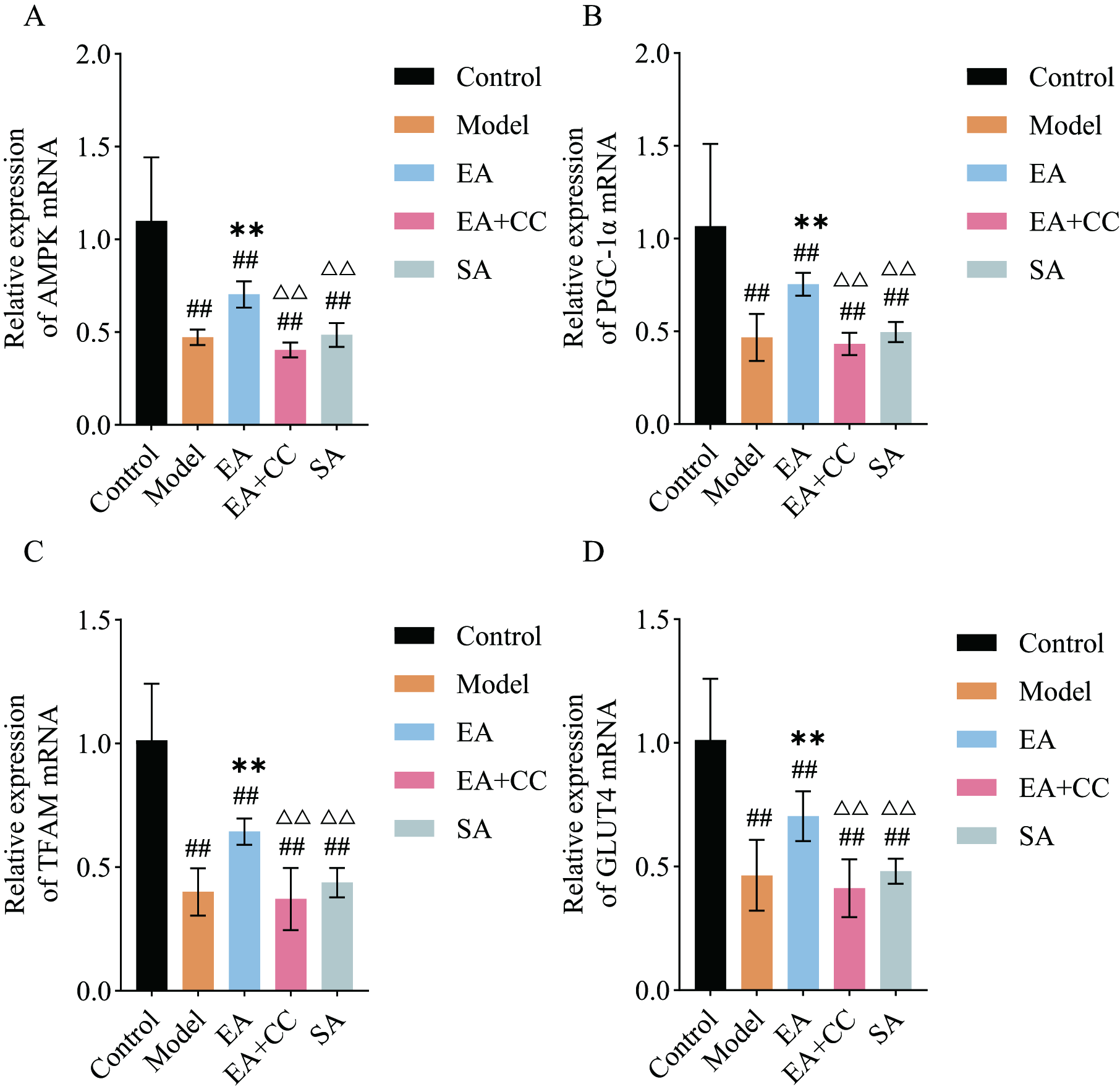

Supplement: Supplementary file 1 — Supplementary Material 1. [file 13098_2025_1960_MOESM1_ESM.zip › table and figure/AMPK mRNA.tif]

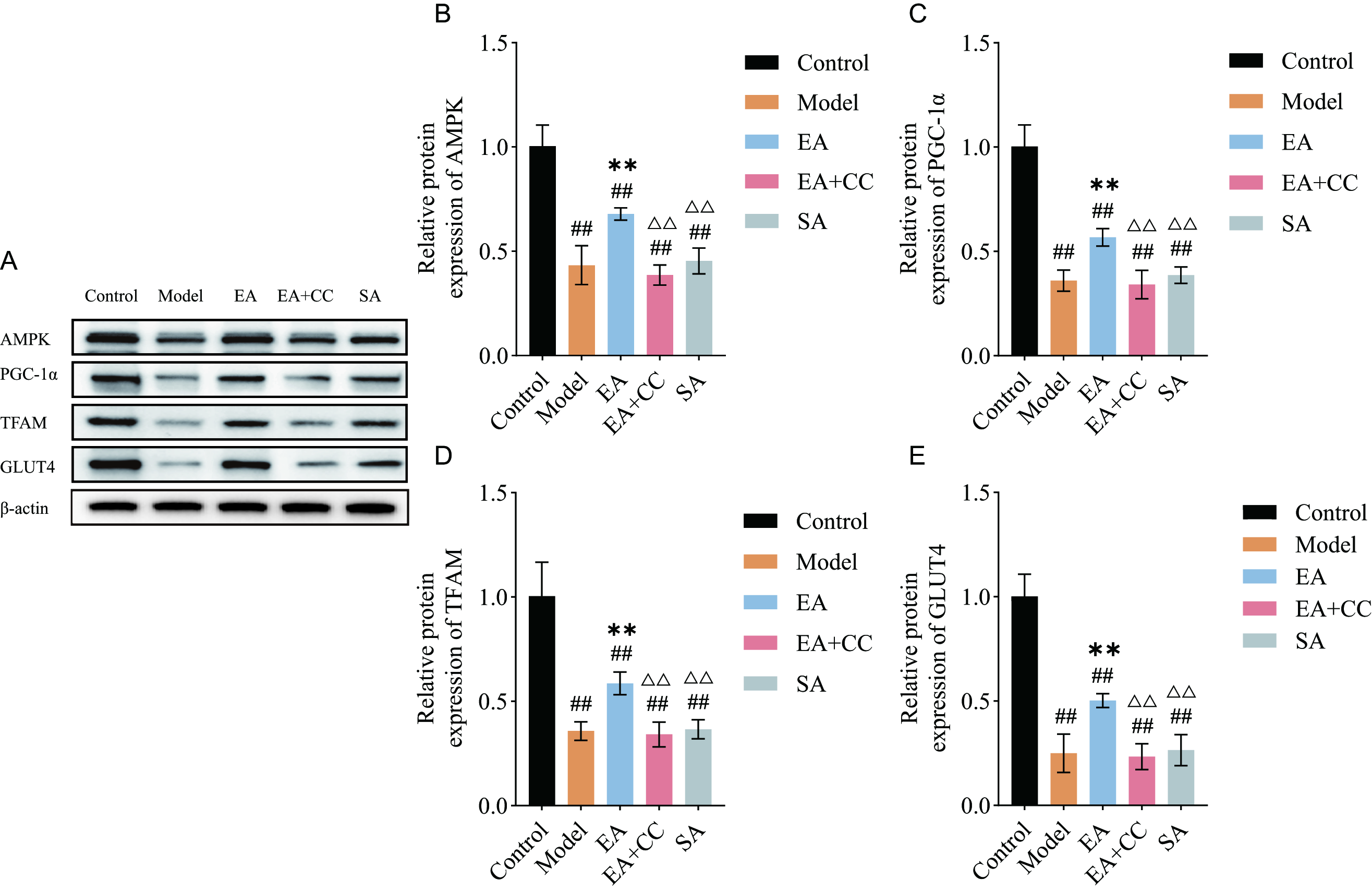

Supplement: Supplementary file 1 — Supplementary Material 1. [file 13098_2025_1960_MOESM1_ESM.zip › table and figure/AMPK.tif]

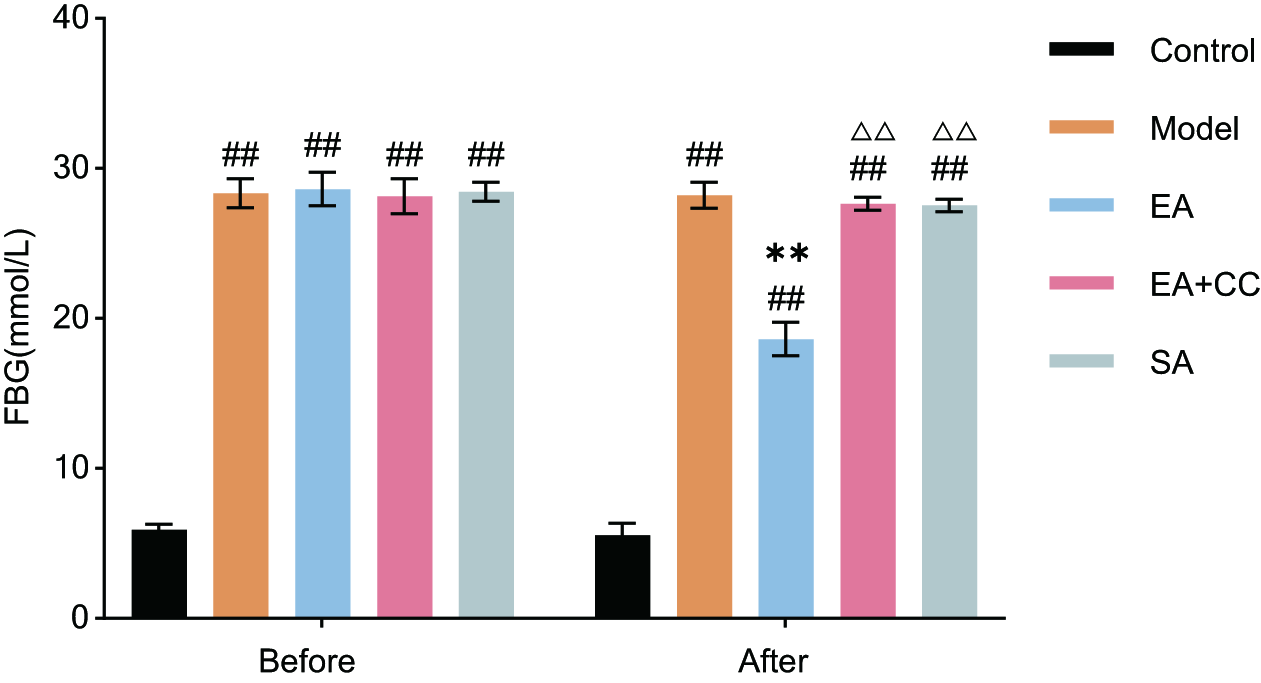

Supplement: Supplementary file 1 — Supplementary Material 1. [file 13098_2025_1960_MOESM1_ESM.zip › table and figure/FBG.tif]

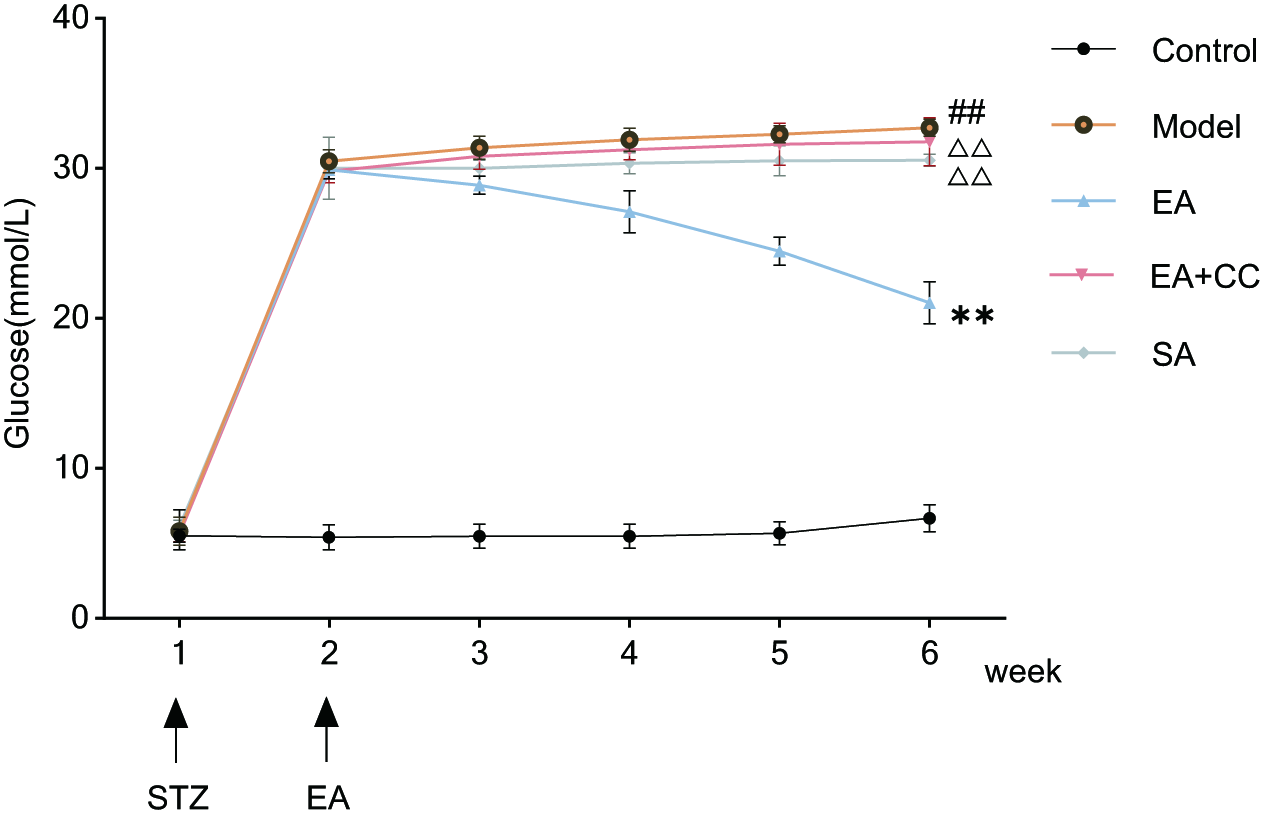

Supplement: Supplementary file 1 — Supplementary Material 1. [file 13098_2025_1960_MOESM1_ESM.zip › table and figure/glucose.tif]

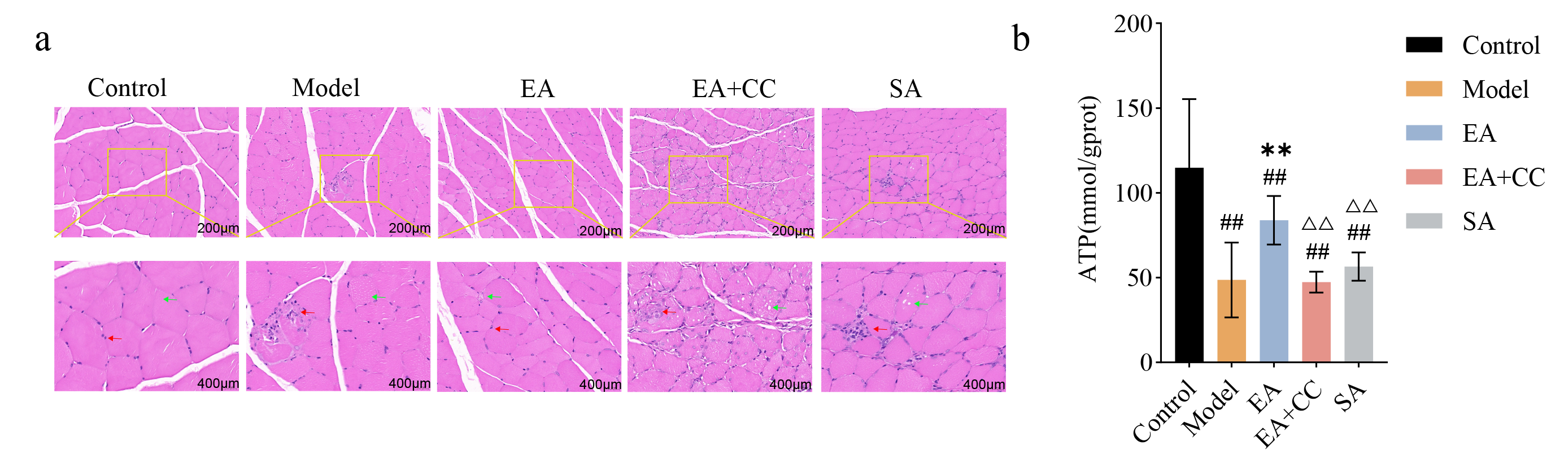

Supplement: Supplementary file 1 — Supplementary Material 1. [file 13098_2025_1960_MOESM1_ESM.zip › table and figure/HE.tif]

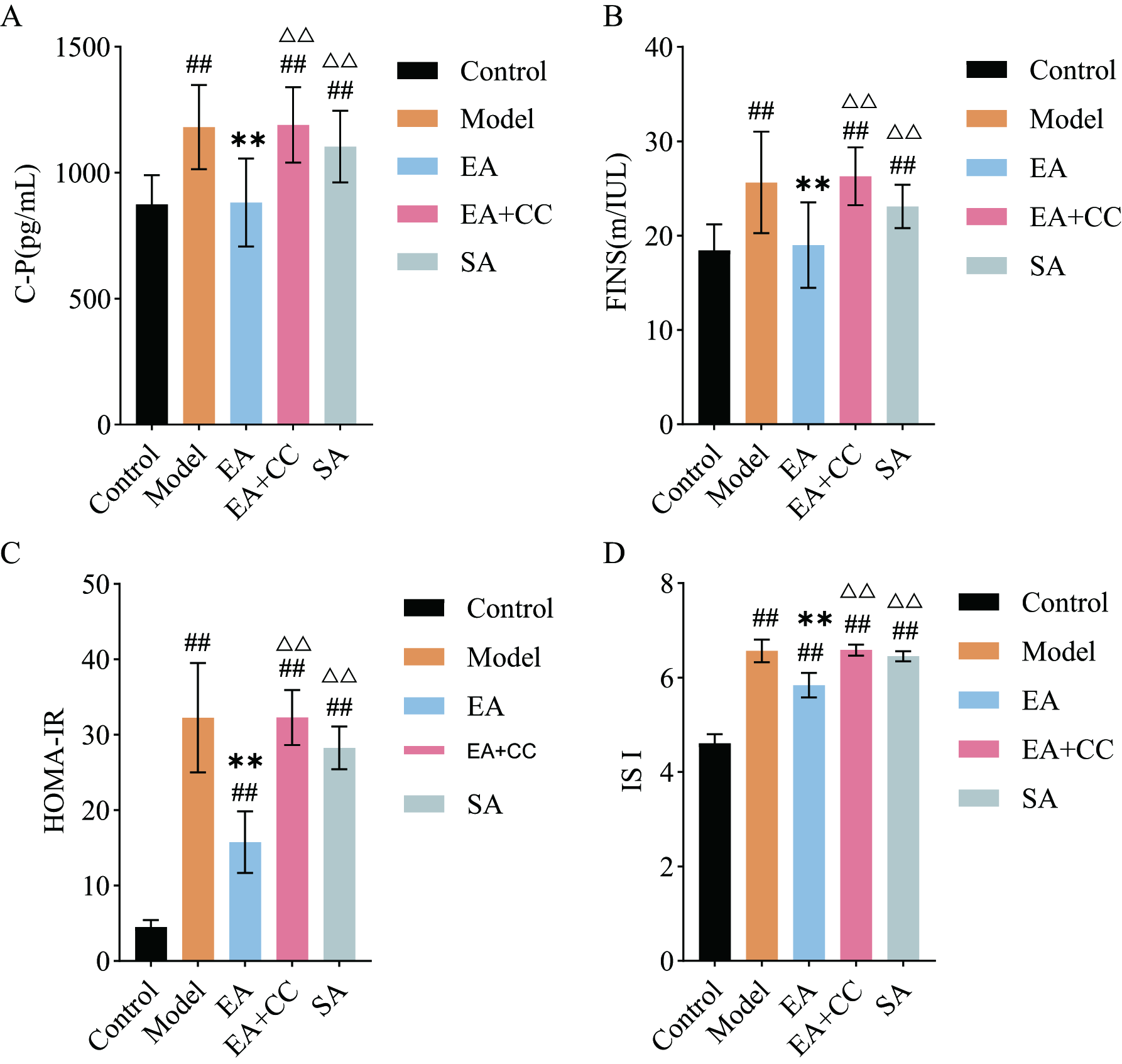

Supplement: Supplementary file 1 — Supplementary Material 1. [file 13098_2025_1960_MOESM1_ESM.zip › table and figure/insulin.tif]

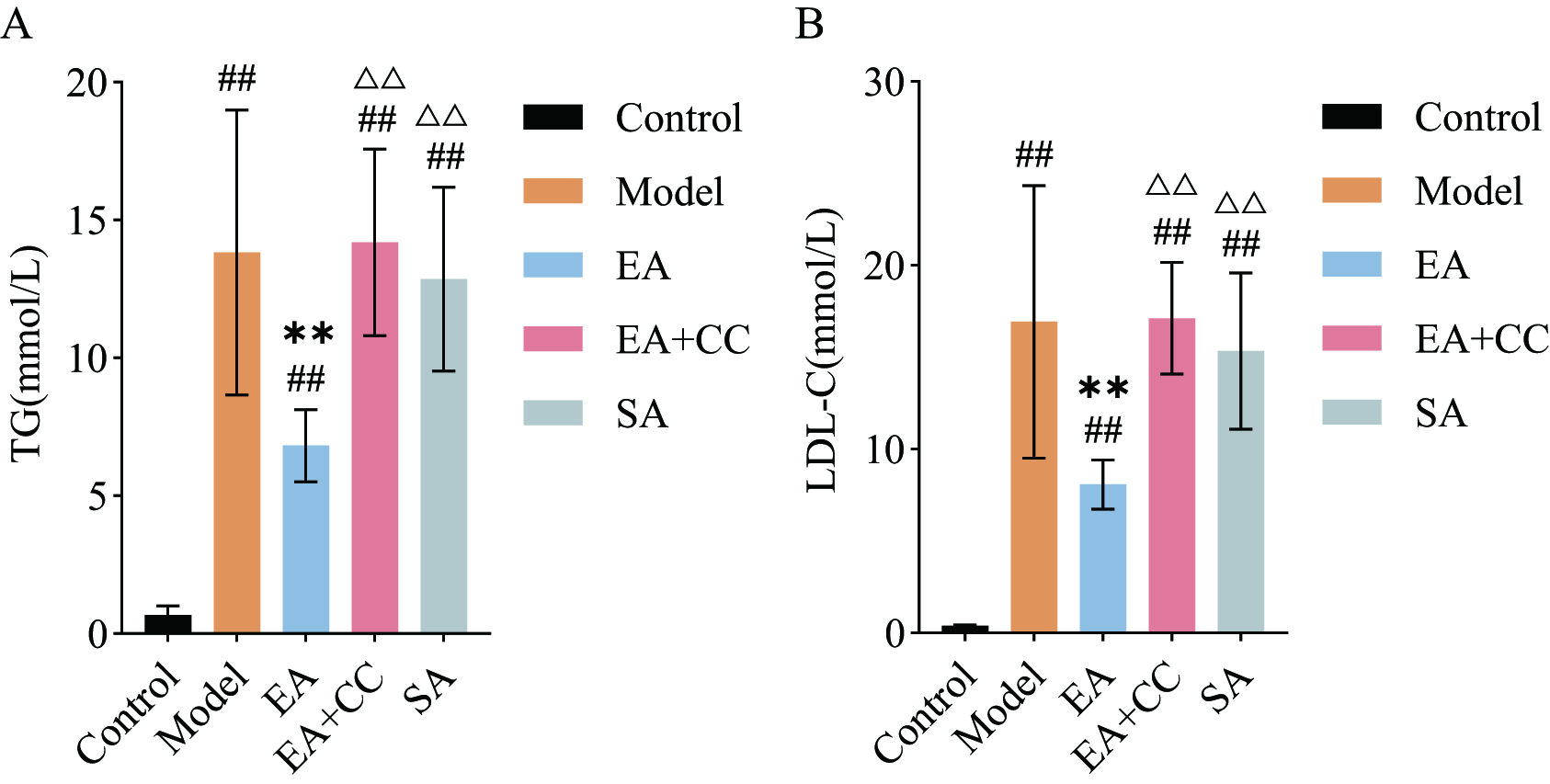

Supplement: Supplementary file 1 — Supplementary Material 1. [file 13098_2025_1960_MOESM1_ESM.zip › table and figure/lipid.tif]

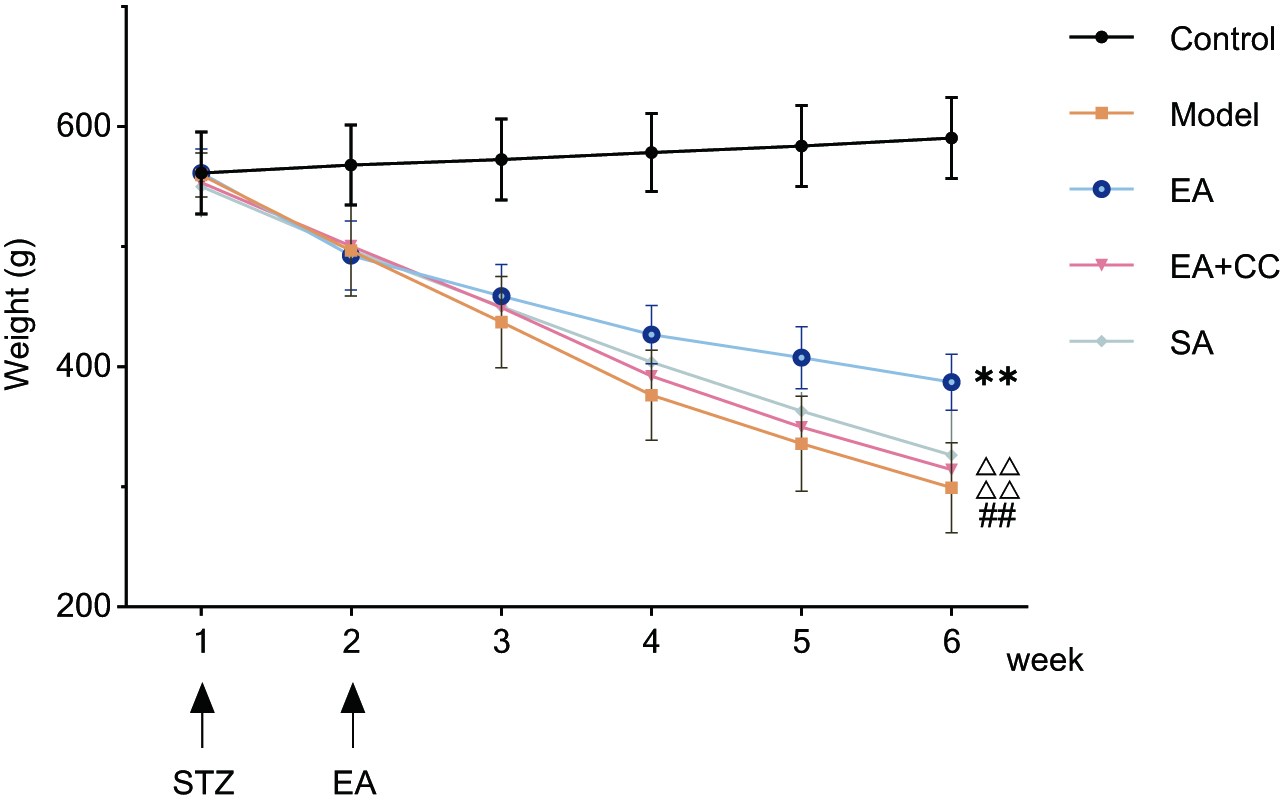

Supplement: Supplementary file 1 — Supplementary Material 1. [file 13098_2025_1960_MOESM1_ESM.zip › table and figure/Weight.tif]
